# Modulating transcription through development of semi-synthetic yeast core promoters

**DOI:** 10.1371/journal.pone.0224476

**Published:** 2019-11-05

**Authors:** Thomas Decoene, Sofie L. De Maeseneire, Marjan De Mey

**Affiliations:** 1 Centre for Synthetic Biology (CSB), Ghent University, Ghent, Belgium; 2 Centre for Industrial Biotechnology and Biocatalysis (InBio.be), Ghent University, Coupure links, Ghent, Belgium; University of Cambridge, UNITED KINGDOM

## Abstract

Altering gene expression regulation by promoter engineering is a very effective way to fine-tune heterologous pathways in eukaryotic hosts. Typically, pathway building approaches in yeast still use a limited set of long, native promoters. With the today’s introduction of longer and more complex pathways, an expansion of this synthetic biology toolbox is necessary. In this study we elucidated the core promoter structure of the well-characterized yeast *TEF1* promoter and determined the minimal length needed for sufficient protein expression. Furthermore, this minimal core promoter sequence was used for the creation of a promoter library covering different expression strengths. This resulted in a group of short, 69 bp promoters with an 8.0-fold expression range. One exemplar had a two and four times higher expression compared to the native *CYC1* and *ADH1* promoter, respectively. Additionally, as it was described that the protein expression range could be broadened by upstream activating sequences (UASs), we integrated earlier described single and multiple short, synthetic UASs in front of the strongest yeast core promoter. This approach resulted to further variation in protein expression and an overall promoter library spanning a 20-fold activity range and covering a length from 69 bp to maximally 129 bp. Furthermore, the robustness of this library was assessed on three alternative carbon sources besides glucose. As such, the suitability of short yeast core promoters for metabolic engineering applications on different media, either in an individual context or combined with UAS elements, was demonstrated.

## Introduction

The yeast *Saccharomyces cerevisiae* serves as an ideal platform organism for the economically viable production of bulk and fine chemicals [1,2]. This however requires the introduction of heterologous metabolic pathways and the fine-tuning of gene expression to find an optimal balance within the production pathway, and between the host’s native metabolism and the imbedded pathway. One effective way to alter and optimize metabolic pathways in yeast is gene expression regulation at the level of transcription. Typically, the two main control elements in eukaryotic transcription are a gene’s promoter and its terminator. Terminators play an important role in controlling mRNA half-life, which has an important influence on the enzyme output levels. Given their decisive role, native expression-enhancing terminators have been intensively characterized and synthetic terminators improving heterologous gene expression have been developed [3–5]. Promoters on the other hand also have a very large impact on gene expression levels and are as such one of the most important parts of the yeast synthetic biology toolbox [6]. A selective group of native yeast promoters is broadly used [7–9], typically representing constitutive and inducible promoters. Commonly used constitutive promoters ensuring gene expression in all conditions are the *TEF1*, *TDH3*, *CYC1* and *ADH1* promoters [10]. Inducible promoters on the other hand allow controllable expression and are activated when desired. Regularly used are the *GAL* and *CUP1* promoters, induced by galactose and copper respectively [6]. In general, constitutive promoters are preferred due to some inherent disadvantages of inducible promoters, such as lag time after induction, leaky expression and potential high inducer costs or inducer toxicity.

**Fig 1:**
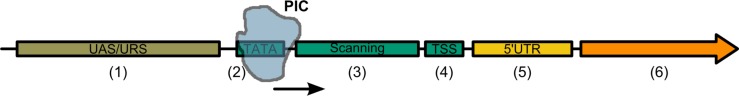
Schematic representation of the structure of a yeast promoter. The yeast promoter is typically divided in upstream activating or repressive sequences (UAS/URS, 1) and the core promoter region (2, 3 and 4). The pre-initiation complex (PIC) containing RNA polymerase II is recruited to the TATA box or a TATA-like sequence (2) and scans (3) the core promoter to a suitable transcription start site (TSS, 4). Other depicted elements are the 5’ untranslated region (5’UTR, 5) and the coding sequence (6).

The structure of eukaryotic promoters is well studied and they are generally divided in a core promoter element and upstream regulatory elements (Fig 1) [11]. The 5’UTR sequence is usually located after the core promoter and plays a major role in the eukaryotic translation initiation process [12–15]. The core promoter is the regulatory sequence to which RNA polymerase II binds and where transcription is started [16–20]. Therefore, it is seen as a major determinant of gene expression in yeast [17]. The length of the core promoter is typically around 100–200 bp and contains a nucleosome free region to enhance access of the pre-initiation complex (PIC) to the DNA. The PIC typically binds to the consensus TATA box and scans the core promoter in search for a suitable transcription start site (TSS) [17]. Despite only 20% of the yeast genes contains a canonical TATA box [21], weaker TATA-like sequences differing up to 2 bp with the consensus are found in almost all yeast promoters and serve as binding sites for the PIC [16]. Though some TSS consensus sequences have been suggested, *i*.*e*. RRYRR, TCRA, YAWR and A(A_rich_)_5_NYAWNN(A_rich_)_6_, to date no fixed TSS sequence in yeast has been agreed on [16]. Generally, transcription is initiated 40 to 120 bp further downstream of the TATA box or the TATA-like sequence in case of TATA-less promoters [16,17,22–24]. Core promoter activity was also observed to be higher with a pyrimidine rich scanning region and an adenine enriched initiation region [17]. Upstream regulatory elements are placed in front of the core promoter and typically contain one or more transcription factor binding sites (TFBSs). These *cis*-acting regulatory DNA stretches recruit transcription factors (TFs) interacting with one another and with the basal transcriptional systems to regulate promoter activity [11]. As such, TFs can be repressors or activators of transcription and bind either to their respective upstream repressive sequence (URS) or upstream activating sequence (UAS) [25]. Promoter engineering strategies by both modulating the core promoter and upstream regulatory DNA elements are thus very effective ways to alter a gene’s expression and hence to balance biosynthetic pathways [26].

Many approaches for promoter engineering in yeast have already been developed [6,11]. Well-known pioneering examples altering constitutive gene expression are error-prone PCR, hybrid promoter engineering and nucleosome affinity modulation. Error-prone PCR involves the introduction of random mutations in an existing promoter sequence. This strategy led for example to a 15-fold range promoter library of the popular *TEF1* promoter [27,28]. In the hybrid approach, the core promoter and UASs are seen as modular building blocks where a core promoter can be combined with one or multiple (different) UASs to alter total promoter activity [26,29]. Lastly, specific mutations suggested by predictive models decreased the nucleosome affinity in promoter sequences and resulted in a more open promoter structure, improving the access of TFs and thus enhancing transcription [30]. Besides modifying the native transcriptional machinery by mutagenesis, the introduction of heterologous and synthetic inducible TFs in front of yeast (core) promoters also showed to be very adequate in controlling gene expression levels [31–37]. Promoters with a high variance within and across different experiments are less interesting as these have a lower predictability and make their use in heterologous pathways unfavorable. This can lead to unreliable product titers which is far from optimal for use in industrial contexts. As such, this makes the robustness and reproducibility of promoters an important element in today’s strain engineering and synthetic biology [38,39]. Therefore, the use of heterologous regulatory parts has the advantage of not interfering with the host’s native cellular regulation, *i*.*e*. orthogonality [37]. In addition, the fact that these TFs can be induced (*e*.*g*. LexA-ER-AD TFs [31,33] and synthetic TFs based on dCas9 and TALEs [34]), ensures the tightly control of (non-native) production pathways, even in different environmental conditions. Likewise, hybrid promoter engineering by varying the number of synthetic TFBSs for inducible TFs in front of the core promoter was very effective to amplify the transcriptional output of the gene of interest [36,37].

Despite these promising reports though, the synthetic biology field of *S*. *cerevisiae* is still hampered by the big length of its promoters. Indeed, native promoters in yeast typically span ranges of hundreds of nucleotides, needed for the recruitment of the large RNA polymerase II. Even when only the core promoter regions are considered in the construction of yeast synthetic circuits, the lengths still vary between 150 and 200 base pairs [34,37]. Together with the fact that every gene in eukaryotes needs its own promoter and terminator, the construction of large biosynthetic pathways in yeast quickly becomes a laborious task. One way to solve this bottleneck could be the use of short, viral promoters such as the bacteriophage T7 promoter having a length of around 20 bp. Although the *in vivo* production of RNA transcripts with this orthogonal system has been demonstrated in *S*. *cerevisiae* [40,41], it has some inherent disadvantages like the requirement of a heterologous expressed T7 RNA polymerase and the inability of translating T7 transcripts due to the lack of a 7-methylguanosine cap which is necessary to initiate translation. Therefore, the construction of short promoters that interact with the native yeast RNA polymerase II is preferred. In addition, since most available and characterized yeast promoters used today in production pathways are based on native sequences [7,8], the occurrence of homologous recombination between the different regulatory elements within the heterologous pathway and with the genome is likely and finally will lead to strain instability. Homologous recombination between parts is also a risk when multiple direct repeats of TFBSs are used, for example to adjust the strength of gene expression [31,34]. It is therefore recommended to use TFBSs differing in nucleotide sequence as much as possible [37]. The fact on the other hand that a lot of described synthetic TFs need to be induced [31–34] could also be seen as a disadvantage. Even though inducible systems allow very precisely control of genetic circuits, the costly inducers make such systems rather elusive in industrial environments.

These hurdles could be tackled by the design of short, constitutive synthetic yeast promoters with a range of different strengths comparable to those of broadly used native constitutive promoters [8]. Currently, the library with the shortest synthetic promoters reported is the one of Redden *et al*. [42], with a length of around 120 bp. Preferably, they should be less than 100 nucleotides in length for easy incorporation in primers, enabling fast transcription unit (TU) construction via PCR. As such, this study describes the development and characterization of a set of short constitutive yeast core promoters. We first identified by way of truncation the minimal length needed for transcription initiation of the well-characterized *TEF1* core promoter. Next, a library of semi-synthetic yeast core promoters (< 70 bp) was constructed by randomization of this *TEF1* minimal core promoter. Finally, this expression range was further expanded by the insertion of existing synthetic upstream activating elements in front of the strongest core promoter [42]. More specifically, these UASs are recognized by native yeast TFs without the need of any induction and have different sequences to exclude any chance of homologous recombination.

## Material and methods

Unless otherwise stated, all products were purchased from Sigma-Aldrich (Diegem, Belgium), all fragments were PCR purified using the innuPREP PCRpure Kit (Analytik Jena AG, Jena, Germany), Circular Polymerase Extension Cloning (CPEC) [43] was used for the assembly of all plasmids and plasmid extraction was performed with the innuPREP Plasmid Mini Kit (Analytik Jena AG).

### Strains and media

*S*. *cerevisiae* SY992 (*Matα*, *ura3Δ0*, *his3Δ1*, *leu2Δ0*, *trp1-63*, *ade2Δ0*, *lys2Δ0*, *ADE8*, Euroscarf, University of Frankfurt, Germany [44]) was used as yeast expression host. All yeast strains derived from this strain are listed in Table A in S1 File. Yeast cultures were grown in synthetic defined (SD) medium consisting of 0.67% YNB without amino acids, a carbon source and selective amino acid supplement mixture without uracil (CSM–URA, MP Biomedicals, Brussel, Belgium). Unless otherwise stated, 2% glucose (Cargill, Sas van Gent, The Netherlands) was the preferred carbon source for SD medium (SDG). Alternative carbon sources used were fructose (SDF, Cargill), pyruvate (SDP) or glycerol (SDGlyc, Chem-lab Analytical, Zedelgem, Belgium) with an equimolar C-content as 2% glucose. To solidify media, 2% Agar Noble (Difco, Erembodegem, Belgium) was added.

Transformax^™^ EC100^™^ Electrocompetent *Escherichia coli* (Lucigen, Halle-Zoersel, Belgium) was used for cloning procedures and for maintaining plasmids. *E*. *coli* strains were cultured in Lysogeny Broth (LB) consisting of 1% tryptone-peptone (Difco), 0.5% yeast extract (Difco), 1% sodium chloride (VWR, Leuven, Belgium) and 100 μg/ml ampicillin. For solid growth medium, 1% agar (Biokar diagnostics, Pantin Cedex, France) was added.

### Plasmid construction

**Fig 2:**
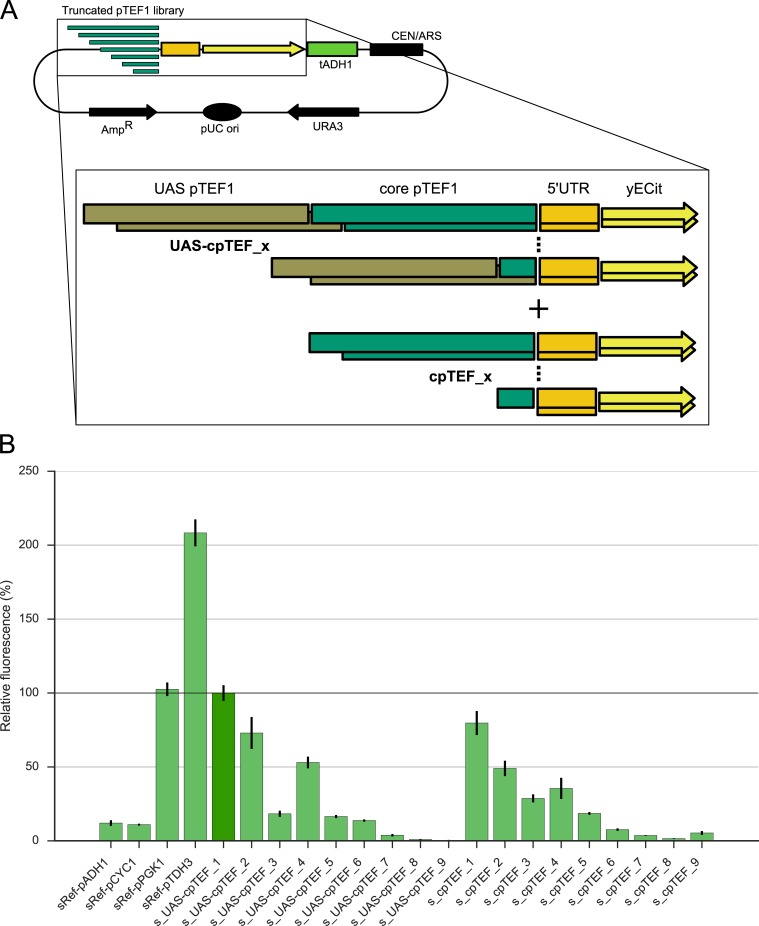
Truncated *TEF1* core promoter library. (A) Schematic overview of the *TEF1* core promoter libraries with (UAS-cpTEF_x) and without (cpTEF_x) the *TEF1* UAS; x varies from 1 to 9. The sequence of the truncated *TEF1* core promoters is given in Table D in S1 File. (B) yECitrine fluorescence obtained with the truncated *TEF1* core promoter libraries and four reference promoters. The values are given relative to s_UAS-cpTEF_1 (dark green, horizontal line) representing the native *TEF1* promoter (sRef-pTEF1). Error bars represent the standard error of the mean (n = 4, biological repeats).

For the evaluation of the promoter libraries, five reference vectors with a yeast enhanced Citrine (yECitrine) TU (pKT140, Euroscarf [45]) under the transcriptional control of the *TEF1* [10], *ADH1* [10], *CYC1* [10], *PGK1* [7] or *TDH3* [10] promoter and the *ADH1* terminator [45] were constructed. yECitrine is a yellow-green fluorescent protein (FP) used a lot as reporter in yeast synthetic biology experiments and has an excitation and emission wavelength around 500 and 530 nm respectively [46]. The TUs were assembled on an in-house low copy yeast expression backbone consisting of a *CEN6/ARS4* origin of replication (ori) and a *URA3* auxotrophic marker (p2a backbone), resulting in the vectors pRef-pTEF1 (S1 Fig), pRef-pADH1, pRef-pCYC1, pRef-pPGK1 and pRef-pTDH3 (Table B in S1 File). All promoter and terminator sequences were picked up from the *S*. *cerevisiae* SY992 genome with PrimeSTAR HS DNA polymerase (Takara, Westburg, Leusden, The Netherlands). In addition, vector pRef-cpRedden1 containing the extra short synthetic core promoter 1 defined by Redden *et al*. [42], was constructed as a reference as well. For its assembly, vector pRef-pTEF1 served as template and the backbone was picked up with overlapping primers 1 and 2 containing the cpRedden1 core promoter sequence (Table C in S1 File). Furthermore, pRef-TEF1 was used as template for the construction of the truncated *TEF1* core promoter library plasmids (Fig 2). The core promoter sequence specified by Blazeck *et al*. [26] was shortened by ca. 20 bp per time through primers 3 to 20 containing overlap sequences for CPEC (Integrated DNA Technologies, Leuven, Belgium, Table C in S1 File and S1 Fig). More specifically, the p2a backbone was split by primers 21 and 22 (Table C in S1 File). Two pieces CPEC, consisting of a part PCR-amplified by the forward core promoter primer (primers 3 to 11 or primers 12 to 20) and primer 22, and a fixed backbone part PCR-amplified by primer 21 and primer 23 or 24, was performed leading to respectively p_UAS-cpTEF_1 to 9 and p_cpTEF_1 to 9.

**Fig 3:**
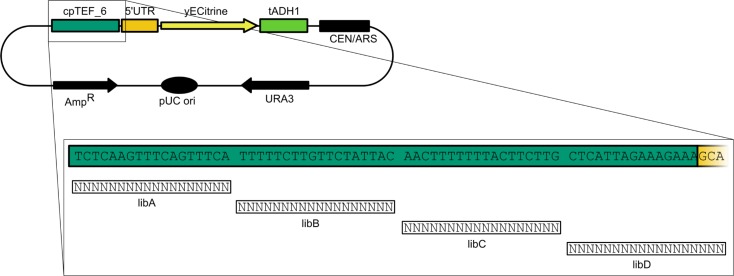
Strategy used for the construction of a minimal *TEF1* core promoter library. Promoter cpTEF_6 was divided into four equal DNA tracts where for every library 18 bp were randomized while the rest of the sequence remained unchanged. Library cpTEF_6-libD randomized also the first three base pairs of the *TEF1* 5’UTR.

Plasmid p_cpTEF_6, containing the 69 bp long minimal *TEF1* core promoter, was used as template for the construction of four *TEF1* core promoter libraries. Four oligonucleotides (primers 25 to 28) each containing 18 degeneracies (IDT, Table C in S1 File) and covering together the whole length of the minimal core promoter were ordered (Fig 3). After plasmid assembly, the distribution of degeneracies (ca. 25% of each nucleotide) was confirmed by Sanger sequencing (EZ-Seq, Macrogen, Amsterdam, The Netherlands).

After screening of the randomized *TEF1* core promoter libraries (see Fluorescence and absorbance measurements), interesting candidates covering a wide range of protein expression were Sanger sequenced (EZ-Seq, Macrogen) to elucidate their sequence. Further, UASs were inserted in front of the strongest obtained core promoter by CPEC. UAS_A_, UAS_C_ and UAS_FEC_ [42] were all placed in front of cpTEF_6-I and cpRedden1, with the latter serving as a reference. As these synthetic upstream elements are very short in length, they could be easily inserted in primers 29 to 40 for high fidelity PCR with p_cpTEF_6-I and p_cpRedden1 as templates (Table C in S1 File).

All plasmids and libraries were transformed in yeast SY992 via the lithium-acetate method [47]. After transformation, strains were selected on SDG–URA plates and confirmed by yeast colony PCR using *Taq* DNA polymerase (NEB, Bioké, Leiden, The Netherlands). For library evaluation, single colonies were randomly picked from agar plate and grown in 96-well microtiter plates (MTPs). An overview of all plasmids used and constructed in this study can be found in Table B in S1 File.

### Fluorescence and absorbance measurements

Fluorescence was used here as a measure of protein levels. Fluorescent proteins are optimized and generally known to fold very well in bacteria and yeasts. As such, it is supposed that these proteins are synthesized in a fully active form and that higher fluorescence levels correspond to more production of the protein. Fluorescence as measure of protein abundance is also widely accepted in the field of synthetic biology [15,26,42,48–50].

Four biological replicates were inoculated from agar plate in sterile 96-well flat-bottomed, black microtiter plates (Greiner Bio-One, Vilvoorde, Belgium) enclosed by a Breathe-Easy^®^ sealing membrane (Sigma-Aldrich) containing 150 μl selective SD–URA medium with the respective carbon source. These plates were incubated on a Compact Digital Microplate Shaker (ThermoFisher Scientific, 3 mm orbit) at 800 rpm and 30°C for 24h. Subsequently, these pre-cultures were diluted 1:150 in 150 μl fresh selective SD–URA medium with the respective carbon source and grown in sterile polystyrene black μclear flat-bottomed 96 well plates (Greiner Bio-One) for evaluation. Except for the randomized *TEF1* core promoter library, the μclear flat-bottomed plate cultures were evaluated in continuous growth experiments performed in a TECAN Infinite^®^ 200 PRO MTP reader (Tecan). Optical density (OD, 600 nm) and fluorescence (FP, excitation and emission of yECitrine, 500 nm and 540 nm, respectively) were measured every 15 min for minimum 50 hours at 30°C (orbital shaking at 2 mm orbit). For every strain, the endpoint OD was determined as the OD value after which three descending OD values were observed. This endpoint OD with its corresponding FP value were used for further data analysis. For the evaluation of the randomized *TEF1* core promoter library, an endpoint OD and FP measurement was taken after 26h of growth (stationary phase) at 30°C while shaking at 800 rpm (Compact Digital Microplate Shaker, 3 mm orbit).

For analysis of fluorescence measurements, two types of controls were included in every single MTP. A medium blank (*i*.*e*. SD–URA medium) was used for the correction of background absorbance of the medium (OD_bg_). sRef-bl lacking fluorescent protein expression and containing p2a_empty was used to correct for the background fluorescence of yeast (FP_bg_). Fluorescence corrected for OD was used as measure for fluorescent protein expression and calculated as follows:

$${\left(\frac{FP}{OD}\right)}_{corrected}=\frac{FP-{FP}_{bg}}{OD-{OD}_{bg}}$$ (1)

The relative fluorescence was defined as follows:

$$Relative\;fluorescence\;(\%)=\frac{{\left(\frac{FP}{OD}\right)}_{corrected}}{{\left(\frac{FP}{OD}\right)}_{corrected,\;ref}}\times 100$$ (2)

### Data analysis

Calculations were performed in Python using the Python Data Analysis Library (Pandas). One-way ANOVA, executed in MS Excel, was used to determine intervariability between promoters in the different growth experiments on glucose (Note: since fluorescence is not an absolute measure, all data was therefore normalized to cpTEF_6 for every experiment). Error bars represent the standard error of the mean (n = 4). Pairwise comparisons between different strains were done by a two-sided T-test using the scipy.stats package in Python. In all cases, a significance level of 0.05 was applied.

## Results and discussion

### The minimal TEF1 core promoter

For the development of minimal yeast core promoters, a yECitrine based fluorescent reporter system to evaluate the altered promoter influence on gene expression was constructed. As a starting point, the *Saccharomyces cerevisiae TEF1* promoter, which is well described in literature [27,28] and is used a lot in yeast synthetic biology approaches [8,51–55], was used. Since the *TEF1* promoter has no canonical TATA box, the distinction between UAS and core promoter is not straightforward. However, based on the categorization of Blazeck *et al*. [26], the *TEF1* promoter was divided in an UAS of 203 bp and a 176 bp long core promoter which starts with the weaker TATA-like sequence AATAAAAA differing in only 1 nucleotide with the TATA box consensus sequence TATA(A/T)A(A/T)(A/G) [21]. Furthermore, this core promoter is followed by a 5’UTR of 33 bp [56,57]. In this study, the 176 bp long core promoter served as template in the search of a minimal promoter sequence which was determined by truncation of the 176 bp core promoter in steps of ca. 20 bp toward its 5’UTR. In addition, the effect of the native UAS on minimal core promoter activity was investigated by developing two sets of truncated promoters, one with and one without the UAS. As such, two libraries of nine promoters with different lengths were constructed (Fig 2A).

Truncating the *TEF1* core promoter led to an overall decrease in protein expression for both libraries (Fig 2B), which was expected since the large RNA polymerase II complex needs a long stretch of DNA for binding and stabilization [58]. However, the truncation of cpTEF_3 to cpTEF_4, which resulted in the complete deletion of a poly-dT stretch, caused an increase in promoter activity. More specifically, from cpTEF_4 on, a complete stretch of 66 nucleotides with 60.6% thymines was deleted compared to the original *TEF1* core promoter. It is reported that such long stretches of consecutive similar nucleotides have an influence on nucleosome affinity. Especially the complement stretch, *i*.*e*. a poly-dA tract, disfavors nucleosome formation and thus promotes binding of regulatory promoter elements which enhances total promoter activity [59–62]. In addition, poly-dA:dT tracts drive nucleosome positioning in the promoter by creating boundaries against which nucleosomes are located [63]. It was indeed observed that nucleosome organization was dramatically changed when a poly-dA:dT tract with its upstream promoter region was deleted [63]. In general, poly-dA:dT tracts are very influential parts crucial for accurate nucleosome organization and thus playing a determining role in the structure of a yeast promoter. This makes them also interesting targets to modify transcriptional regulation of gene expression. Hence, we suggest that this poly-dT removal in the *TEF1* core promoter could be a major cause of the sudden increase in yECitrine fluorescence. It is also noteworthy that the truncated library led to very short promoters with activities higher than native long and weak yeast promoters. For example, cpTEF_5 existing of only 90 bp was 1.5-fold stronger than the 1500 bp long *ADH1* promoter. This confirms (minimal) core promoters as key determinants of gene expression levels [17].

Pairwise comparisons of the UAS-cpTEF_x and cpTEF_x strains showed only a significant positive influence of the UAS on protein expression for strain s_UAS-cpTEF_6 (p = 6.73E-4) and a significant negative effect for strains s_UAS-cpTEF_3 and s_UAS-cpTEF_9 (p = 0.013 and p = 0.008, respectively). This was somewhat surprising since the addition of an UAS_TEF1_ in front of a truncated *LEU* promoter [26] significantly increased expression levels. However, when taking in mind that the p-values of strains 1, 2 and 4 were very close to the significance level (p ≈ 0.05) and that others report similar results to ours in that respect that an extra *CIT1* or *CLB2* UAS in front of some synthetic core promoter elements also did not improve protein expression [42], our results are in line with earlier observations. For the shortest core promoter elements, UAS_TEF1_ does not influence expression levels, presumably by the inability of RNA polymerase II to bind, leading to complete failure of proper transcription initiation.

Altogether, since cpTEF_6 is the shortest core promoter giving rise to detectable transcription (*i*.*e*. ca. 0.66-fold lower than the weak *ADH1* and *CYC1* promoters) and showing significant activation by its UAS, this core promoter element was chosen for random library construction.

### Random TEF1 core promoter library

For the construction of a range of short core promoters in *S*. *cerevisiae*, a randomization approach with degenerated oligo’s spanning the 69 bp long core promoter cpTEF_6 was used (Fig 3). Both to allow us to capture positional effects of the randomization and to sample significant quantities of the created variance, cpTEF_6 was divided into four DNA stretches of 18 base pairs. One stretch per library was degenerated, whereas the others were kept equal to the native sequence. To ensure for every library the same space of possibilities, cpTEF_6-libD was extended with the first three base pairs of the *TEF1* 5’UTR. Evaluation of these four libraries could give an idea of the regions in cpTEF_6 that have a high influence on protein expression and are interesting for further analysis. For example, a similar approach in evaluating the effect of mutations in the *AOX1* core promoter of *Pichia pastoris* showed that the TATA-box, the region downstream of the TSS and the region near to the start codon had a significant influence on gene expression [64]. Furthermore, a library of 7536 unique 118 bp long core promoter sequences gave a thorough insight in the regions that significantly affect core promoter strength in *S*. *cerevisiae* [17]. The main disadvantage of our randomization approach to obtain strong core promoters compared to the concept of Redden *et al*. [42], is the fact that our short core promoters are not fully synthetic. Especially for the reason that homologies of 20 to 30 bp are already sufficient to initiate homologous recombination in *S*. *cerevisiae*. Nevertheless, homologies of 60 bp or more are needed for highly efficient homologous recombination which is not the case for our promoters having a native part of around 50 bp.

**Fig 4:**
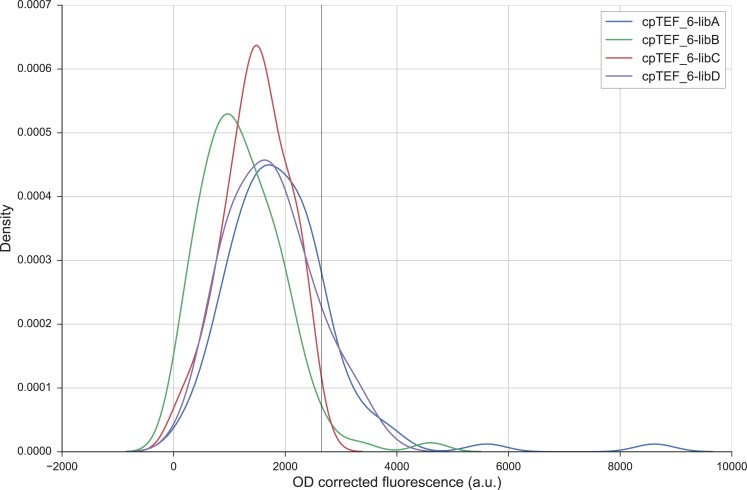
Histograms of the four randomized cpTEF_6 libraries. For 94 randomly chosen colonies, yECitrine fluorescence was analyzed. The vertical line represents the mean fluorescence (2645 ± 332.7 a.u., n = 4, biological repeats) of the native cpTEF_6 promoter.

From each library 94 colonies were randomly picked and evaluated for fluorescence (*i*.*e*. yECitrine). sRef-bl and s_cpTEF_6 were taken along as references in every MTP. Histograms of the four libraries revealed a small shift toward higher fluorescence levels for libraries cpTEF_6-libA and D (Fig 4, S2–S5 Figs, p-values when comparing the means for library A and D to library C and B at least < 0.01). Both library A and D gave thus more rise to promoters with higher strengths than the native cpTEF_6 compared to library B and C (12 and 13 versus 2 and 0). This indicates that both regions in the cpTEF_6 promoter are interesting to enhance transcription levels. Library D, varied immediately in front of the 5’UTR, including in the TSS, confirms that the region around the TSS is an important feature for initiation of mRNA transcription. It has indeed been described that the scanning of RNA polymerase II in search of a suitable TSS depends on its surrounding context [17,65–67]. A comparable result was also seen in the *AOX1* core promoter of *P*. *pastoris*, where mutations downstream the TSS affected eGFP fluorescence [64]. Library A, varied at the 5’ end of the cpTEF_6 promoter sequence, is positioned around 50 nucleotides away from the TSS. It has been reported that transcription in *S*. *cerevisiae* is started 40 to 120 bp downstream of the TATA box or a TATA-like sequence (in the case of pTEF1) and variation in this region alters core promoter activity [16]. As such, novel TATA boxes or TATA-like sequences are possibly generated in library A of the minimal core promoter, making it the ideal position to bind and compose the PIC and to subsequently start transcription. Libraries cpTEF_6-libB and C are suggested to form a scanning region of around 40 bp between the PIC binding place and the TSS and they seem to generate more promoters with lower activity. This is plausible as the native cpTEF_6 spacer region is already enriched in T and C (∼ 80%) and a T/C-rich scanning region was linked with higher expression levels [17].

**Fig 5:**
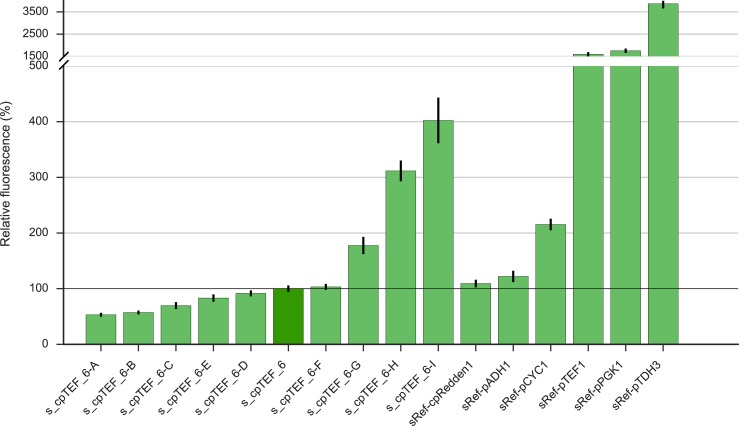
Characterization of nine selected promoters from library cpTEF_6-libA. Protein expression levels were normalized against the native cpTEF_6 promoter (dark green, horizontal line). Error bars represent the standard error of the mean (n = 4, biological repeats). All strains are listed in Table A in S1 File.

As cpTEF_6-libA had a high potential to contain more high-expressing core promoters (*e*.*g*. promoters with OD corrected fluorescence over 5000 a.u. were reached, Fig 4), 281 additional colonies were randomly selected and analyzed together with three biological repeats of s_cpTEF_6 and sRef-bl. Analysis of fluorescence levels revealed a similar distribution pattern as for the first 94 colonies analyzed (S6 Fig). Again, some very high expressing core promoters were identified compared to the native sequence, a result which was not achieved by error-prone PCR of the whole *TEF1* promoter [28]. From all the screened colonies of cpTEF_6-libA, four stronger and five weaker promoters covering the whole expression range were sampled and used for further characterization. The promoter activity of this range of nine 69 bp long core promoters was compared to five commonly used native yeast promoters, *i*.*e*. pADH1, pCYC1, pTEF1, pPGK1 and pTDH3, and to the core promoter cpRedden1, which is one of the shortest yeast core promoters currently reported [42] (Fig 5). Interestingly, the results show that we obtained three promoters (s_cpTEF_6-G, H and I) having a 2.0 to 4.0-fold higher strength than the native cpTEF_6 minimal core promoter (s_cpTEF_6) we started from. When looking into detail in these three sequences, a clear appearance of a TATA-like sequence differing in only 1 or 2 nucleotides from a real consensus TATA box (TATA(A/T)A(A/T)(A/G) [21]) is visible (Table D in S1 File). Such TATA-like sequences could not be determined in the weaker s_cpTEF_6 core promoters. This is in line with the findings of Lubliner *et al*. (2015) where it was observed that core promoters having a TATA box or a TATA-like sequences gave rise to higher gene expression levels [17]. Also in other yeasts like *P*. *pastoris*, a profound effect in promoter functionality was observed by mutating the TATA box [64]. In addition, while using a promoter sequence of only 69 bp, cpTEF_6-G, H and I ranged from equal to double expression levels compared to the weak *CYC1* (287 bp) promoter. Also, these three promoters are stronger than the yeast *ADH1* promoter, having a length of 1461 bp. Compared to the core promoter reported by Redden *et al*. (sRef_cpRedden1), having a length of around 65 bp, our truncated cpTEF_6 had equal yECitrine levels. Since the lengths of both core promoters are very similar, this could indicate the minimal stretch of DNA needed for proper transcription initiation in yeast. Furthermore, after randomization in cpTEF_6, core promoters transcending the strength of cpRedden1 were retrieved, even without upstream elements. Despite the fact that the native pTEF1 is still four times stronger than the short cpTEF_6-I, the latter does have a reduction of 82% in sequence length. As such, the reported set of promoters (Fig 5 and Table D in S1 File) shows that an adequate expression range (8-fold) can be achieved using core promoter elements smaller than 70 bp and lacking any UASs.

Although a set of nine minimal core promoters has been described before [42], the set described in this study consists of short promoters, *i*.*e*. 69 bp, that are stronger than for example the native *CYC1* promoter (up to 2 times) without the need of UASs. In earlier observations by Redden *et al*., an extra UAS had to be added to the core promoters to reach higher expression levels than the native *CYC1* promoter, leading to promoters over the 100 bp in length [42]. Additionally, this reduction in length without huge loss of promoter activity enlarges the modularity of introducing yeast promoters in TUs via PCR. Together with the usage of short terminators [4], this implies yeast TUs can now be assembled in one single PCR with the coding sequence of interest, whereas with native regulatory elements these parts must be picked up first before assembly in a TU.

Still, the obtained promoter library of short core promoters shows a less broader expression range compared to the earlier reported 15-fold *TEF1* promoter library [28]. The main difference to our approach is that Nevoigt and coworkers varied the whole 412 bp long *TEF1* promoter (*i*.*e*. core promoter inclusive its UAS). As such, not only the sequence to bind RNA polymerase II is altered, but also the sequence to recruit transcription factors which could consequently lead to extra variation in gene expression. Therefore, in the view to further broaden our expression range, the influence of introducing extra UAS sequences in front of the strongest yeast core promoter cpTEF_6-I was evaluated.

### Expanding the expression range with synthetic UASs

In a next step, we applied hybrid promoter engineering to obtain stronger promoters and broader expression ranges. Hybrid promoter engineering, with UAS elements serving as modular amplifiers in front of a core promoter, has proven to be an effective technique for the enhancement of gene expression levels in yeast [26,29]. Also in front of synthetic minimal core promoters, adding extra UASs further enhanced promoter strength to a range comparable of the strongest yeast promoters [42]. In view of minimizing the total promoter length, three previously described, fully synthetic UAS elements of only 10 bp were selected: UAS_A_, UAS_C_ and UAS_FEC_ with the latter in fact an array of three synthetic UAS elements (Table E in S1 File) [42]. The semi-synthetic cpTEF_6-I promoter, the strongest core promoter retrieved in this study, was chosen as proof of principle to assess the potential of amplifying its promoter strength by UASs. As a reference, all chosen UAS elements were also placed in front of cpRedden1. Since these synthetic UASs are only 10 bp in length, the assembly of these constructs is strongly facilitated. More specifically, their sequence can be incorporated in primer extensions to pick up core promoter parts which can be afterwards assembled in expression vectors or integrated in the genome. In this study, CPEC [43] was preferred as assembly technique to quickly introduce the different UASs in front of the core promoters.

**Fig 6:**
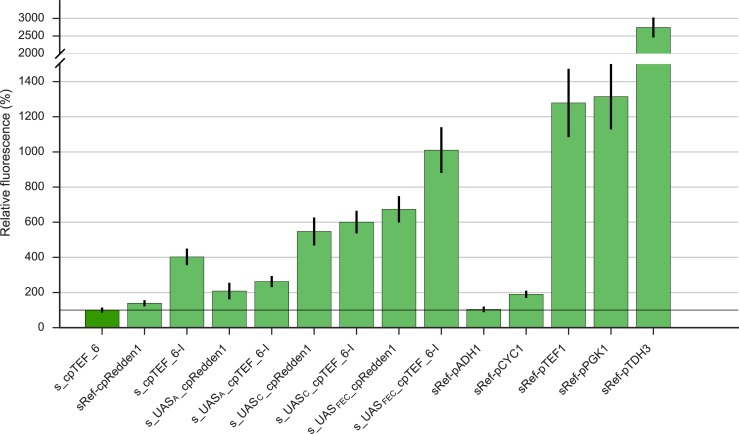
Overview of the effect on yECitrine expression of single (UAS_A_, UAS_C_) and multiple UASs (UAS_FEC_). All UASs were placed in front of yeast core promoter cpTEF_6-I. As a reference, all UASs were also placed in front of cpRedden1. Protein expression levels were normalized against the native cpTEF_6 promoter (dark green, horizontal line). Error bars represent the standard error of the mean (n = 4, biological repeats). All strains are listed in Table A in S1 File.

The effect of the different UAS elements on transcription initiation was evaluated by measuring yECitrine fluorescence on SDG medium (Fig 6). To start with, it is noteworthy to mention that fluorescence levels obtained with UAS_A_ have some divergent effects. Where expression is controlled by core promoter cpRedden1, UAS_A_ has a significant amplifying impact (1.4 times, p = 0.019) which was also seen in earlier observations [42]. Conversely, this upstream element had a repressive effect when combined with cpTEF_6-I (p = 8.6E-5) since fluorescence levels were almost halved. The fact that the same UAS in front of different core promoters could have some different efficiencies was earlier demonstrated with the UAS of the mitotic cyclin gene *CLB2* [42]. On the other hand, UAS_C_ and UAS_FEC_ both significantly increased the promoter activity for cpRedden1 by a factor 3.9 and 4.8 and for cpTEF_6-I by a factor 1.5 and 2.5, respectively (p-values at least < 2.4E-5). In all cases, stronger promoters than the reference promoters with cpRedden1 were achieved, however the impact of UASs on the increase in promoter activity was smaller for cpTEF_6-I. These results reveal that the degree of change in transcription levels is strongly dependent on the combination of a specific UAS type with a specific core promoter. Furthermore, multiple UAS elements, *i*.*e*. UAS_FEC_, can certainly be used as modular building blocks to amplify transcription, but the magnitude of their effect is difficult to predict. This confirms an important interplay between UASs and core promoters where both must be compatible for the enhancement of transcription [25,68].

Nevertheless, UAS_FEC__cpTEF_6-I led overall to the highest expression, which was even a factor 10 larger than the cpTEF_6 we initially started from. Even, this promoter, with a total promoter length of only 129 bp, showed almost a comparable activity to the native and strong yeast *TEF1* and *PGK1* promoters. Considering these are bulky promoters with lengths of respectively 379 [10] and 1257 bp [7], nearly an equal expression outcome was reached in solely 34% and 10% of the total promoter length. As such, the total length of transcription units could be drastically decreased. However, creating a promoter with strengths comparable to the strongest yeast promoter pTDH3 was not possible. The yECitrine expression under control of UAS_FEC__cpTEF_6-I stranded at roughly 33%, yet it has a reduction of 80% in DNA load. These observations are partially in agreement with the study of Redden *et al*. since their final promoter also contributed to strong gene expression in only 20% of the pTDH3 DNA space [42]. Despite, a promoter with a strength up to 70% of pTDH3 was reached which is double as high as in our measurements. Modifying the 5’UTR sequence to obtain higher translation initiation rates [12–15] could further help here to obtain expression profiles similar to the strong pTDH3. Nevertheless, the aim of obtaining stronger promoters using hybrid promoter engineering was reached, as, with the exception of UAS_A__cpTEF_6-I, all hybrid promoters obtained were stronger than the cpTEF_6-I promoter. In general, this study delivered a semi-synthetic promoter library that spans an overall expression range on glucose medium between about 53% and 1000% of the original 69 bp cpTEF_6 promoter.

With view on the accuracy of promoters across the different growth experiments on SDG medium (Fig 2, Fig 5 and Fig 6), the variation of the reference yeast promoters and the most promising promoter cpTEF_6-I was also evaluated. One-way ANOVA revealed that only the native yeast promoters pTEF1 and pADH1 (p-value of 0.11 and 0.10, respectively) and our novel semi-synthetic core promoter cpTEF_6-I (p = 0.98) led to reproducible fluorescence levels between the independent growth experiments (Table F in S1 File). Especially all other tested native promoters (*i*.*e*. pCYC1, pPGK1 and pTDH3) showed a lower chance of equal activities under the same conditions (p-values at least < 9.15E-3). In this regard, pTEF1 and pADH1 are trustworthy promoters on glucose for strong and weak gene expression respectively and are preferred above pPGK1 and pCYC1 having comparable strengths.

### Promoter evaluation on different carbon sources

Since it is known that the activity of yeast promoters can change on different carbon sources [69,70], synthetic promoters can be seen as a valuable alternative for the creation of robust hosts with reproducible transcriptional output, and substantially constant product titers. In this view, the robustness of the semi-synthetic promoter library was assessed on synthetic defined fructose, pyruvate and glycerol medium. Comparable to glucose, fructose is a fermentable carbon source directly entering the glycolytic pathway, while pyruvate and glycerol are non-sugar carbon sources entering the cell via alternative pathways which often lead to varied regulation at the transcriptional level [71].

In general, promoter activity on fructose (S7 Fig) resembles very well its activity on glucose which seems logical as both substrates enter the glycolysis at the beginning. As such, it is less likely that other pathways needed for proper growth are activated. The strong native yeast promoters on pyruvate, which is the end product of the glycolysis, showed reduced yECitrine levels relatively toward cpTEF_6 (*e*.*g*. 1500% compared to 3000% for pTDH3 on glucose, S8 Fig). The fact that the glycolysis, where normally pTDH3 and pPGK1 are highly activated, is not necessarily needed here to form pyruvate could be a main explanation for this phenomenon. Nevertheless, the activity range of the semi-synthetic promoters (cpTEF_6-A to I) on SDP medium where comparable when growing on SDG medium. Only for UAS_A_ and UAS_C_, the amplifying effect on promoter strength for cpTEF_6-I and cpRedden1 disappeared on pyruvate (S8 Fig), which is probably due to a different activation of transcription factors when using non-sugar substrates [71]. The largest difference in activity for the semi-synthetic promoters was seen on glycerol (S9 Fig). cpTEF_6-I was only 2.5 times stronger of cpTEF_6 while this promoter was up to 4 times stronger on the other media, and remarkably, all promoters of Redden *et al*. [42] led to very low expression levels which was not observed on the other media. Also here, the addition of UAS_A_ and UAS_C_ did not have a significant effect on yECitrine expression implying a different mechanism of transcription factor activation when grown on non-sugar carbon sources.

Despite these subtle differences in expression strengths, the selected weak and strong promoters from the semi-synthetic library on glucose (cpTEF_6-A to I) had in general an equal behavior on the alternative media (Fig 5 and Fig 6 vs. S7 Fig, S8 Fig and S9 Fig). For example, cpTEF_6-A was always one of the weakest and cpTEF_6-H was always one of the strongest promoters independent from the medium. In addition, synthetic core promoter cpTEF_6-I and its derivatives led in all cases to the highest gene expression compared to the other members of the library. As a result, UAS_FEC__cpTEF_6-I can be seen as an absolute short and robust promoter alternative for pTEF1 and pPGK1. Since synthetic promoters are less subjected to intracellular regulation, this library could as such be seen as an ideal tool for pathway engineering in industrial yeast production strains under different environmental conditions.

## Conclusion

In this study, the well-characterized *TEF1* promoter was truncated to elucidate its minimal core promoter and to enable the design of short functional yeast promoters. Six of the nine truncated promoters remained functional, even without the addition of an UAS and a 69 bp long *TEF1* minimal core promoter, cpTEF_6, was determined. Randomization of this core promoter sequence revealed influential regions at its 5’ and 3’ ends, respectively suggesting a location of the PIC region and confirming the importance of the region around the TSS. This randomization approach led to short semi-synthetic yeast promoters with a very reproducible output (*cfr*. cpTEF_6-I) compared to commonly used native promoters. Furthermore, an increasing number of UASs in front of this core promoter gradually enhanced transcription, however, no stronger protein expression could be achieved as with the pTDH3 which is less good compared to similar work by Redden *et al*. where 70% was reached of the total pTDH3 strength [42]. On the other hand, our work revealed very short core promoters (~70 bp) with expression profiles in line of pCYC1 and pADH1, and outperforming the strengths of the core promoters from Redden *et al*. [42], even when using different carbon sources besides glucose. Accordingly, the strongest promoters in both studies had a length of roughly 120–130 bps. This indicates it is almost impossible to attain promoters existing of less than 120 nucleotides and having similar performances of the strongest yeast promoters. Therefore, the combination with other regulatory parts like (synthetic) terminators [3,4], also affecting transcription, and 5’UTRs [12–15], which influence translation initiation, also needs consideration in transcription unit design. In future work, incorporating upstream elements and core promoters in standardized Golden Gate vectors could further contribute to a plug-and-play assembly system comparable to VEGAS [72]. By doing so, different UASs in front of a chosen core promoter can be quickly combined to obtain (short) yeast promoters with a desired expression strength. Also investigating the genomic stability of the semi-synthetic core promoters will be necessary as stable regulatory parts are a critical requirement for metabolic engineering applications.

Altogether, this study demonstrated the possibility of short yeast promoter libraries, of which the expression range can be expanded with UASs, to function as full transcriptional regulators. As a result, a usable promoter library validated in four different media with lengths not exceeding 130 bp and having a 20-fold expression range is available for yeast metabolic engineering purposes.

## Supporting information

S1 Fig Annotated Genbank file of the UASTEF1-cpTEF_1–5’UTRTEF1-yECitrine-tADH1 transcription unit in pRef-pTEF1 and p_UAS-cpTEF_1. The *TEF1* 5’UTR is indicated in bold and underlined. The UASTEF1 is underlined and the *TEF1* core promoter is indicated in bold. The respective sequences of the truncated *TEF1* core promoter are represented in Table D in S1 File. Primer sites are indicated in yellow and are respectively primers 24, 23, 21 and 22 in order of occurrence. For the p_cpTEF plasmids, the UASTEF1 sequence in front of the core promoter was not present.
(PDF)

S2 Fig Scatter plot of random core promoter library cpTEF_6-libA. The horizontal line represents the mean fluorescence corrected for OD of the native cpTEF_6 which was grown as biological triplicate. Error bars representing the standard error are a consequence of OD correction with biological triplicates of sRef-bl and the medium.
(PDF)

S3 Fig Scatter plot of random core promoter library cpTEF_6-libB. The horizontal line represents the mean fluorescence corrected for OD of the native cpTEF_6 which was grown as biological triplicate. Error bars representing the standard error are a consequence of OD correction with biological triplicates of sRef-bl and the medium.
(PDF)

S4 Fig Scatter plot of random core promoter library cpTEF_6-libC. The horizontal line represents the mean fluorescence corrected for OD of the native cpTEF_6 which was grown as biological triplicate. Error bars representing the standard error are a consequence of OD correction with biological triplicates of sRef-bl and the medium.
(PDF)

S5 Fig Scatter plot of random core promoter library cpTEF_6-libD. The horizontal line represents the mean fluorescence corrected for OD of the native cpTEF_6 which was grown as biological triplicate. Error bars representing the standard error are a consequence of OD correction with biological triplicates of sRef-bl and the medium.
(PDF)

S6 Fig Scatter plot of random core promoter library cpTEF_6-libA for 281 randomly chosen colonies. The horizontal line represents the mean fluorescence corrected for OD of the native cpTEF_6 which was grown as biological triplicate. Error bars representing the standard error are a consequence of OD correction with biological triplicates of sRef-bl and the medium.
(PDF)

S7 Fig Characterization of yeast strains carrying the fluorescence reporter yECitrine under control of various novel synthetic yeast promoters when cultivated in minimal medium with fructose as carbon-source. Protein expression levels were normalized against the native cpTEF_6 promoter (dark green, horizontal line). Error bars represent the standard error of the mean (n = 4, biological repeats). All strains are listed in Table A in S1 File.
(PDF)

S8 Fig Characterization of yeast strains carrying the fluorescence reporter yECitrine under control of various novel synthetic yeast promoters when cultivated in minimal medium with pyruvate as carbon-source. Protein expression levels were normalized against the native cpTEF_6 promoter (dark green, horizontal line). Error bars represent the standard error of the mean (n = 4, biological repeats). All strains are listed in Table A in S1 File.
(PDF)

S9 Fig Characterization of yeast strains carrying the fluorescence reporter yECitrine under control of various novel synthetic yeast promoters when cultivated in minimal medium with glycerol as carbon-source. Protein expression levels were normalized against the native cpTEF_6 promoter (dark green, horizontal line). Error bars represent the standard error of the mean (n = 4, biological repeats). All strains are listed in Table A in S1 File.
(PDF)

S1 File Supplemental tables. Table A. Strains used in this study. Table B. Plasmids used in this study. Table C. Primers used in this study. Table D. Sequences of the core promoters. Table E. Synthetic upstream activating sequences. Table F. p-values obtained after one-way ANOVA to investigate the yECitrine variability of the native and synthetic promoters.
(PDF)
