# Supplementary material for: Modulating transcription through development of semi-synthetic yeast core promoters
Source: PLoS One. 2019 Nov 5;14(11):e0224476. doi: 10.1371/journal.pone.0224476 (PMC6830820; doi:10.1371/journal.pone.0224476)
Supplement: S1 Fig — The TEF1 5’UTR is indicated in bold and underlined. The UASTEF1 is underlined and the TEF1 core promoter is indicated in bold. The respective sequences of the truncated TEF1 core promoter are represented in Table D in S1 File. Primer sites are indicated in yellow and are respectively primers 24, 23, 21 and 22 in order of occurrence. For the p_cpTEF plasmids, the UASTEF1 sequence in front of the core promoter was not present. (PDF) [file pone.0224476.s001.pdf]

```
LOCUS       pRef-pTEF1       5940 bp    DNA     circular SYN 17-DEC-2017
DEFINITION  Join_product added to end of PCR_prod_p2a_111_VBB, overlap trimmed
ACCESSION   p2a111_EcoRI-TEF
KEYWORDS    .
SOURCE      Unknown.
  ORGANISM  Unknown
            Unclassified.
REFERENCE   1  (bases 1 to 5940)
  AUTHORS   Self
  JOURNAL   Unpublished.
COMMENT     SECID/File created by Clone Manager, Scientific & Educational Software
FEATURES             Location/Qualifiers
     misc_feature    complement(146..401)
                     /label=''lacZ
                     /SECDrawAs="Info only"
     misc_feature    402..604
                     /gene="UAS_TEF1"
                     /product="upstream activating sequence TEF1 gene"
                     /SECDrawAs="Region"
                     /SECStyleId=1
     misc_feature    605..813
                     /gene="TEF1 core promoter"
                     /SECDrawAs="Region"
                     /SECStyleId=1
     CDS             814..1530
                     /gene="'yECitrine"
                     /product="yeast enhanced yellow fluorescent protein"
                     /codon_start=3
                     /translation="V"
                     /SECDrawAs="Gene"
                     /SECStyleId=1
                     /SECName="yECitrine"
                     /SECDescr="yeast enhanced yellow fluorescent protein"
     misc_feature    1538..1740
                     /gene="ADH1t"
                     /product="ADH terminator"
                     /SECDrawAs="Region"
                     /SECStyleId=1
     misc_feature    1809..1936
                     /gene="Cen6"
                     /product="yeast chromosome VI centromere sequence"
                     /SECDrawAs="Region"
                     /SECStyleId=1
     misc_feature    1949..2322
                     /gene="ars4"
                     /product="ARS209; histone H4 autonomously replicating
                     sequence"
                     /SECDrawAs="Region"
                     /SECStyleId=1
     misc_feature    2576..2807
                     /gene="pURA3"
                     /SECDrawAs="Region"
                     /SECStyleId=1
     CDS             2808..3611
                     /gene="URA3"
                     /product="orotidine-5'-phosphate decarboxylase"
                     /SECDrawAs="Gene"
                     /SECStyleId=1
     misc_feature    3602..3679
                     /gene="tURA3"
                     /product="URA terminator"
                     /SECDrawAs="Region"
                     /SECStyleId=1
```

```
     misc_feature    4061..4780
                     /gene="pUC ori"
                     /SECDrawAs="Region"
                     /SECStyleId=1
     CDS             complement(4880..5740)
                     /gene="AmpR"
                     /SECDrawAs="Gene"
                     /SECStyleId=1
ORIGIN
        1 tcgcgcgttt cggtgatgac ggtgaaaacc tctgacacat gcagctcccg gagacggtca
       61 cagcttgtct gtaagcggat gccgggagca gacaagcccg tcagggcgcg tcagcgggtg
      121 ttggcgggtg tcggggctgg cttaactatg cggcatcaga gcagattgta ctgagagtgc
      181 accatatgcg gtgtgaaata ccgcacagat gcgtaaggag aaaataccgc atcaggcgcc
      241 attcgccatt caggctgcgc aactgttggg aagggcgatc ggtgcgggcc tcttcgctat
      301 tacgccagct ggcgaaaggg ggatgtgctg caaggcgatt aagttgggta acgccagggt
      361 tttcccagtc acgacgttgt aaaacgacgg ccagtgaatt catagcttca aaatgtttct
      421 actccttttt tactcttcca gattttctcg gactccgcgc atcgccgtac cacttcaaaa
      481 cacccaagca cagcatacta aatttcccct ctttcttcct ctagggtgtc gttaattacc
      541 cgtactaaag gtttggaaaa gaaaaaagag accgcctcgt ttctttttct tcgtcgaaaa
      601 aggcaataaa aatttttatc acgtttcttt ttcttgaaaa tttttttttt tgattttttt
      661 ctctttcgat gacctcccat tgatatttaa gttaataaac ggtcttcaat ttctcaagtt
      721 tcagtttcat ttttcttgtt ctattacaac tttttttact tcttgctcat tagaaagaaa
      781 gcatagcaat ctaatctaag ttttaattac aaaatgtcta aaggtgaaga attattcact
      841 ggtgttgtcc caattttggt tgaattagat ggtgatgtta atggtcacaa attttctgtc
      901 tccggtgaag gtgaaggtga tgctacttac ggtaaattga ccttaaaatt tatttgtact
      961 actggtaaat tgccagttcc atggccaacc ttagtcacta ctttaggtta tggtttgatg
     1021 tgttttgcta gatacccaga tcatatgaaa caacatgact ttttcaagtc tgccatgcca
     1081 gaaggttatg ttcaagaaag aactattttt ttcaaagatg acggtaacta caagaccaga
     1141 gctgaagtca agtttgaagg tgatacctta gttaatagaa tcgaattaaa aggtattgat
     1201 tttaaagaag atggtaacat tttaggtcac aaattggaat acaactataa ctctcacaat
     1261 gtttacatca tggctgacaa acaaaagaat ggtatcaaag ttaacttcaa aattagacac
     1321 aacattgaag atggttctgt tcaattagct gaccattatc aacaaaatac tccaattggt
     1381 gatggtccag tcttgttacc agacaaccat tacttatcct atcaatctgc cttatccaaa
     1441 gatccaaacg aaaagagaga ccacatggtc ttgttagaat ttgttactgc tgctggtatt
     1501 acccatggta tggatgaatt gtacaaataa ggcgcgccac ttctaaataa gcgaatttct
     1561 tatgatttat gatttttatt attaaataag ttataaaaaa aataagtgta tacaaatttt
     1621 aaagtgactc ttaggtttta aaacgaaaat tcttattctt gagtaactct ttcctgtagg
     1681 tcaggttgct ttctcaggta tagtatgagg tcgctcttat tgaccacacc tctaccggca
     1741 cccggggagc gtcccaaaac cttctcaagc aaggttttca gtataatgtt acatgcgtac
     1801 acccgtcggg tccttttcat cacgtgctat aaaaataatt ataatttaaa tttttttaata
     1861 taaatatata aattaaaaat agaaagtaaa aaaagaaatt aaagaaaaaa tagtttttgt
     1921 tttccgaaga tgtaaaagac tctaggggga tcgccaacaa atactacctt ttatcttgct
     1981 cttcctgctc tcaggtatta atgccgaatt gtttcatctt gtctgtgtag aagaccacac
     2041 acgaaaatcc tgtgatttta cattttactt atcgttaatc gaatgtatat ctatttaatc
     2101 tgcttttctt gtctaataaa tatatatgta aagtacgctt tttgttgaaa ttttttaaac
     2161 ctttgtttat ttttttttct tcattccgta actcttctac cttctttatt tactttctaa
     2221 aatccaaata caaaacataa aaataaataa acacagagta aattcccaaa ttattccatc
     2281 attaaaagat acgaggcgcg tgtaagttac aggcaagcga tccgtcctaa gaaaccatta
     2341 ttatcatgac attaacctat aaaaataggc gtatcacgag gccctttcgt ctcgcgcgtt
     2401 tcggtgatga cggtgaaaac ctctgacaca tgcagctccc ggagacggtc acagcttgtc
     2461 tgtaagcgga tgccgggagc agacaagccc gtcagggcgc gtcagcgggt gttggcgggt
     2521 gtcggggctg gcttaactat gcggcatcag agcagattgt actgagagtg caccatacca
     2581 cagcttttca attcaattca tcattttttt tttattcttt tttttgattt cggtttcttt
     2641 gaaatttttt tgattcggta atctccgaac agaaggaaga acgaaggaag gagcacagac
     2701 ttagattggt atatatacgc atatgtagtg ttgaagaaac atgaaattgc ccagtattct
     2761 taacccaact gcacagaaca aaaacctgca ggaaacgaag ataaatcatg tcgaaagcta
     2821 catataagga acgtgctgct actcatccta gtcctgttgc tgccaagcta tttaatatca
     2881 tgcacgaaaa gcaaacaaac ttgtgtgctt cattggatgt tcgtaccacc aaggaattac
     2941 tggagttagt tgaagcatta ggtcccaaaa tttgtttact aaaaacacat gtggatatct
     3001 tgactgattt ttccatggag ggcacagtta agccgctaaa ggcattatcc gccaagtaca
     3061 atttttttact cttcgaagac agaaaatttg ctgacattgg taatacagtc aaattgcagt
     3121 actctgcggg tgtatacaga atagcagaat gggcagacat tacgaatgca cacggtgtgg
     3181 tgggcccagg tattgttagc ggtttgaagc aggcggcaga agaagtaaca aaggaaccta
```

```
3241 gaggcctttt gatgttagca gaattgtcat gcaagggctc cctatctact ggagaatata
3301 ctaagggtac tgttgacatt gcgaagagcg acaaagattt tgttatcggc tttattgctc
3361 aaagagacat gggtggaaga gatgaaggtt acgattggtt gattatgaca cccggtgtgg
3421 gtttagatga caagggagac gcattgggtc aacagtatag aaccgtggat gatgtggtct
3481 ctacaggatc tgacattatt attgttggaa gaggactatt tgcaaaggga agggatgcta
3541 aggtagaggg tgaacgttac agaaaagcag gctgggaagc atatttgaga agatgcggcc
3601 agcaaaacta aaaaactgta ttataagtaa atgcatgtat actaaactca caaattagag
3661 cttcaattta attatatcag ttattaccct atgcggtgtg aaatacggcg taatcatggt
3721 catagctgtt tcctgtgtga aattgttatc cgctcacaat tccacacaac atacgagccg
3781 gaagcataaa gtgtaaagcc tggggtgcct aatgagtgag ctaactcaca ttaattgcgt
3841 tgcgctcact gcccgctttc cagtcgggaa acctgtcgtg ccagctgcat taatgaatcg
3901 gccaacgcgc ggggagaggc ggtttgcgta ttgggcgctc ttccgcttcc tcgctcactg
3961 actcgctgcg ctcggtcgtt cggctgcggc gagcggtatc agctcactca aaggcggtaa
4021 tacggttatc cacagaatca ggggataacg caggaaagaa catgtgagca aaaggccagc
4081 aaaaggccag gaaccgtaaa aaggccgcgt tgctggcgtt tttccatagg ctccgccccc
4141 ctgacgagca tcacaaaaat cgacgctcaa gtcagaggtg gcgaaacccg acaggactat
4201 aaagatacca ggcgtttccc cctggaagct ccctcgtgcg ctctcctgtt ccgaccctgc
4261 cgcttaccgg atacctgtcc gcctttctcc cttcgggaag cgtggcgctt tctcatagct
4321 cacgctgtag gtatctcagt tcggtgtagg tcgttcgctc caagctgggc tgtgtgcacg
4381 aaccccccgt tcagcccgac cgctgcgcct tatccggtaa ctatcgtctt gagtccaacc
4441 cggtaagaca cgacttatcg ccactggcag cagccactgg taacaggatt agcagagcga
4501 ggtatgtagg cggtgctaca gagttcttga agtggtggcc taactacggc tacactagaa
4561 gaacagtatt tggtatctgc gctctgctga agccagttac cttcggaaaa agagttggta
4621 gctcttgatc cggcaaacaa accaccgctg gtagcggtgg tttttttgtt tgcaagcagc
4681 agattacgcg cagaaaaaaa ggatctcaag aagatccttt gatcttttct acggggtctg
4741 acgctcagtg gaacgaaaac tcacgttaag ggattttggt catgagatta tcaaaaagga
4801 tcttcaccta gatcctttta aattaaaaat gaagttttaa atcaatctaa agtatatatg
4861 agtaaacttg gtctgacagt taccaatgct taatcagtga ggcacctatc tcagcgatct
4921 gtctatttcg ttcatccata gttgcctgac tccccgtcgt gtagataact acgatacggg
4981 agggcttacc atctggcccc agtgctgcaa tgataccgcg agacccacgc tcaccggctc
5041 cagatttatc agcaataaac cagccagccg gaagggccga gcgcagaagt ggtcctgcaa
5101 ctttatccgc ctccatccag tctattaatt gttgccggga agctagagta agtagttcgc
5161 cagttaatag tttgcgcaac gttgttgcca ttgctacagg catcgtggtg tcacgctcgt
5221 cgtttggtat ggcttcattc agctccggtt cccaacgatc aaggcgagtt acatgatccc
5281 ccatgttgtg caaaaaagcg gttagctcct tcggtcctcc gatcgttgtc agaagtaagt
5341 tggccgcagt gttatcactc atggttatgg cagcactgca taattctctt actgtcatgc
5401 catccgtaag atgcttttct gtgactggtg agtactcaac caagtcattc tgagaatagt
5461 gtatgcggcg accgagttgc tcttgcccgg cgtcaatacg ggataatacc gcgccacata
5521 gcagaacttt aaaagtgctc atcattggaa aacgttcttc ggggcgaaaa ctctcaagga
5581 tcttaccgct gttgagatcc agttcgatgt aacccactcg tgcacccaac tgatcttcag
5641 catcttttac tttcaccagc gtttctgggt gagcaaaaac aggaaggcaa aatgccgcaa
5701 aaaagggaat aagggcgaca cggaaatgtt gaatactcat actcttcctt tttcaatatt
5761 attgaagcat ttatcagggt tattgtctca tgagcggata catatttgaa tgtatttaga
5821 aaaataaaca aataggggtt ccgcgcacat ttccccgaaa agtgccacct gacgtctaag
5881 aaaccattat tatcatgaca ttaacctata aaaataggcg tatcacgagg ccctttcgtc
//
```

S1 Fig: Annotated Genbank file of the UAS$_{TEF1}$-cpTEF_1-5'UTR$_{TEF1}$-yECitrine-tADH1 transcription unit in pRef-pTEF1 and p_UAS-cpTEF_1. The *TEF1* 5'UTR is indicated in bold and underlined. The UAS$_{TEF1}$ is underlined and the *TEF1* core promoter is indicated in bold. The respective sequences of the truncated *TEF1* core promoter are represented in Table D in S1 File. Primer sites are indicated in yellow and are respectively primers 24, 23, 21 and 22 in order of occurrence. For the p_cpTEF plasmids, the UAS$_{TEF1}$ sequence in front of the core promoter was not present.
