# Supplementary figures and images for: Modulating transcription through development of semi-synthetic yeast core promoters
Source: PLoS One. 2019 Nov 5;14(11):e0224476. doi: 10.1371/journal.pone.0224476 (PMC6830820; doi:10.1371/journal.pone.0224476)

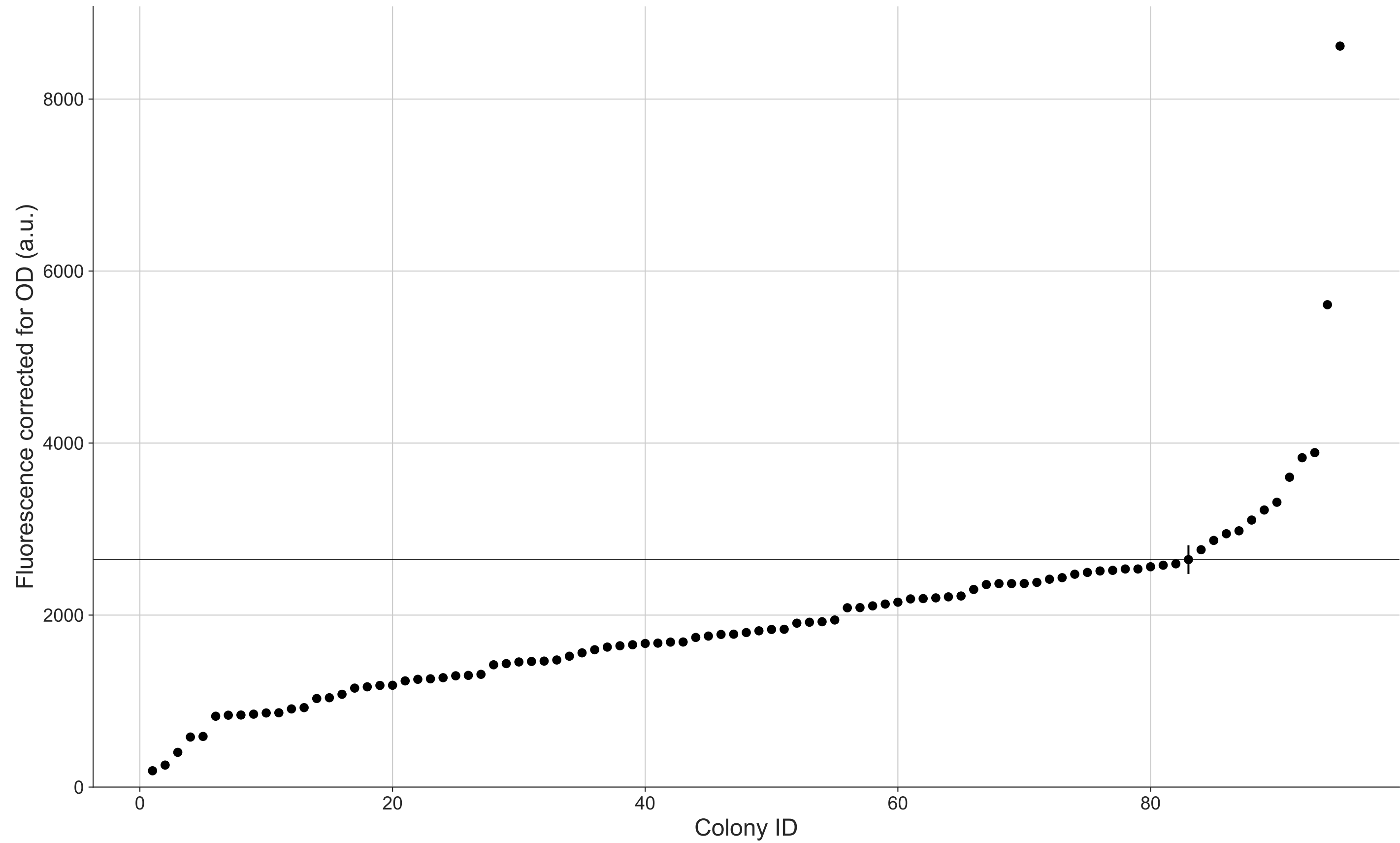

Supplement: S2 Fig — The horizontal line represents the mean fluorescence corrected for OD of the native cpTEF_6 which was grown as biological triplicate. Error bars representing the standard error are a consequence of OD correction with biological triplicates of sRef-bl and the medium. (PDF) [file pone.0224476.s002.pdf]

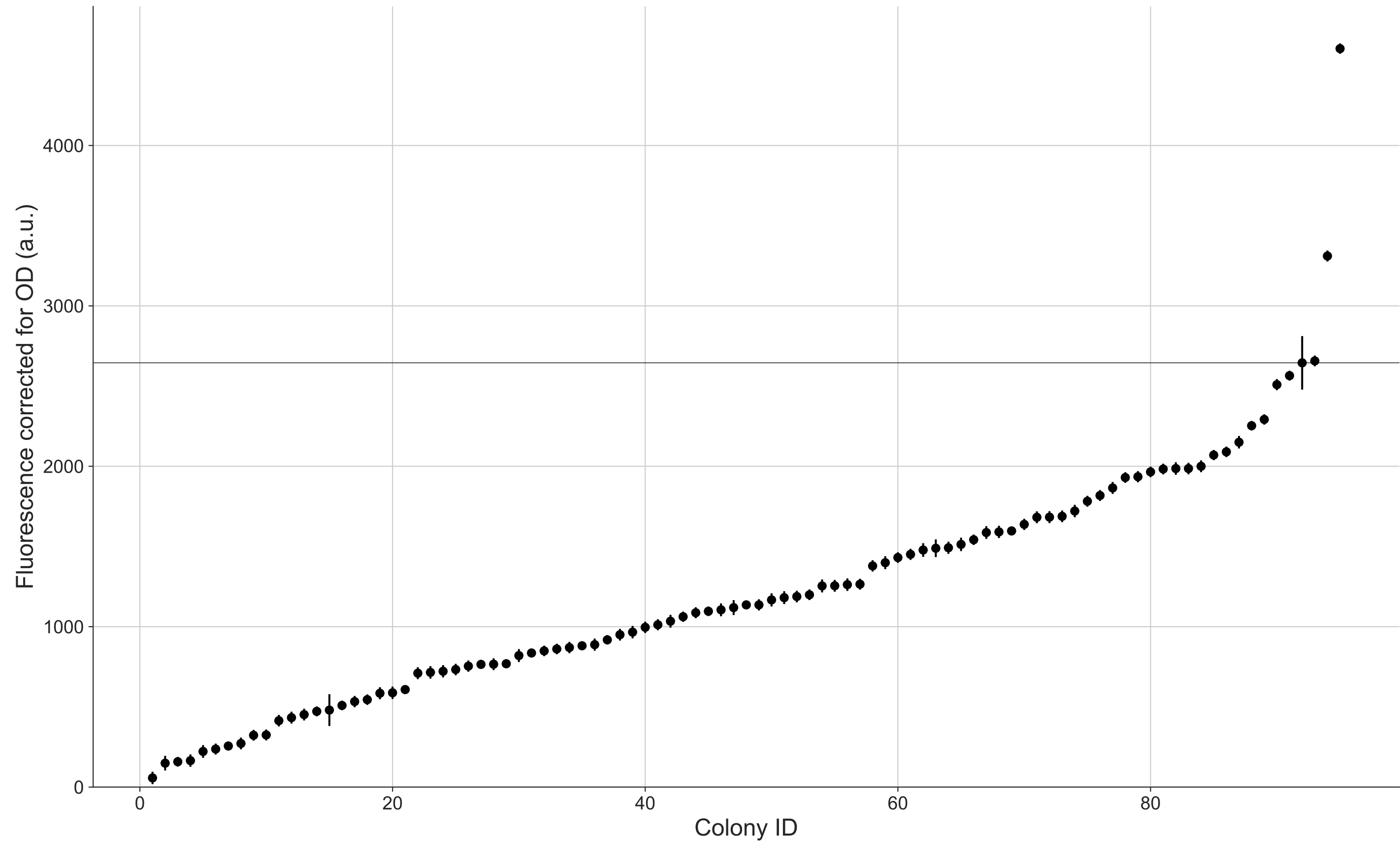

Supplement: S3 Fig — The horizontal line represents the mean fluorescence corrected for OD of the native cpTEF_6 which was grown as biological triplicate. Error bars representing the standard error are a consequence of OD correction with biological triplicates of sRef-bl and the medium. (PDF) [file pone.0224476.s003.pdf]

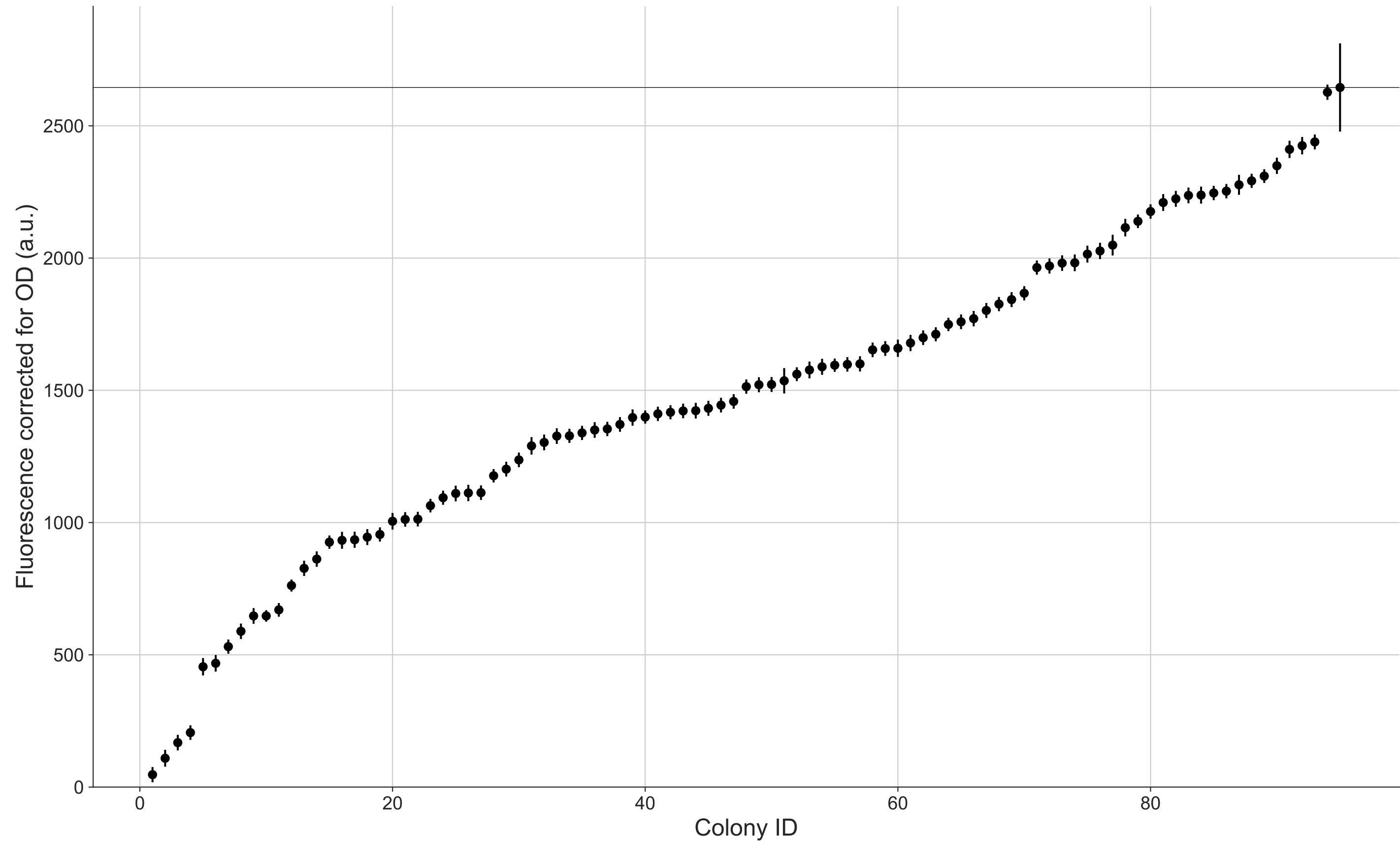

Supplement: S4 Fig — The horizontal line represents the mean fluorescence corrected for OD of the native cpTEF_6 which was grown as biological triplicate. Error bars representing the standard error are a consequence of OD correction with biological triplicates of sRef-bl and the medium. (PDF) [file pone.0224476.s004.pdf]

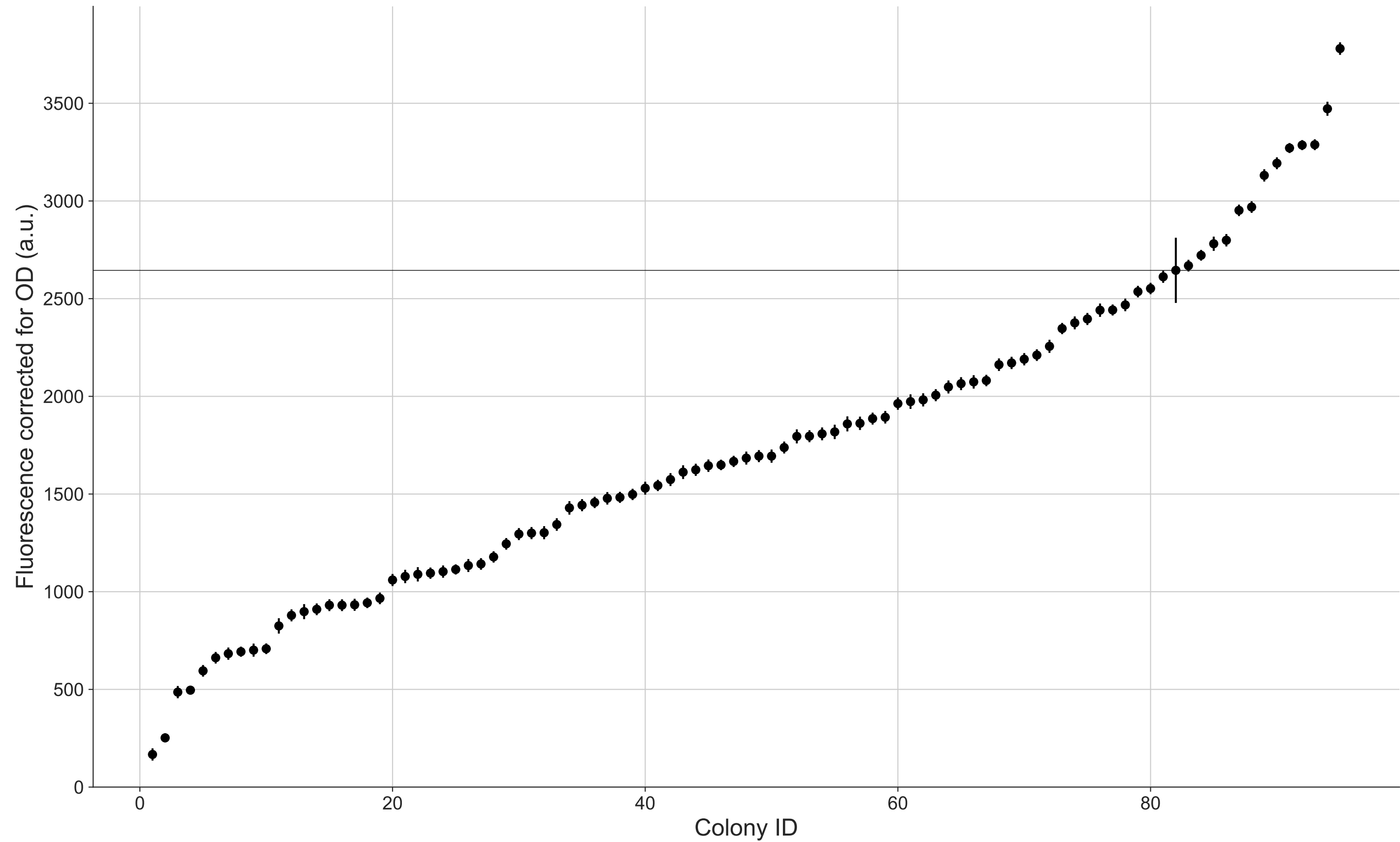

Supplement: S5 Fig — The horizontal line represents the mean fluorescence corrected for OD of the native cpTEF_6 which was grown as biological triplicate. Error bars representing the standard error are a consequence of OD correction with biological triplicates of sRef-bl and the medium. (PDF) [file pone.0224476.s005.pdf]

cpTEF\_6-libA 281 colonies

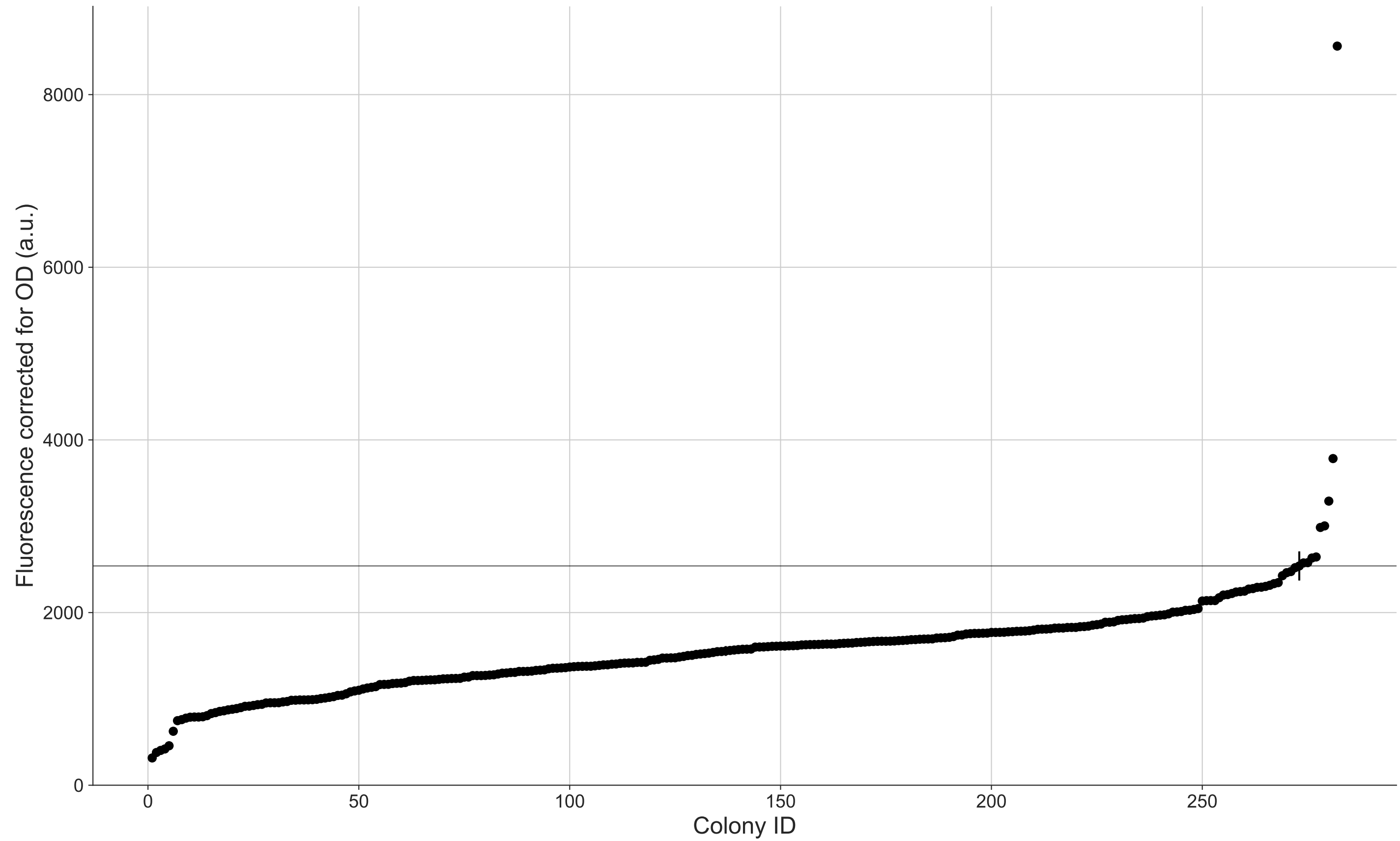

Supplement: S6 Fig — The horizontal line represents the mean fluorescence corrected for OD of the native cpTEF_6 which was grown as biological triplicate. Error bars representing the standard error are a consequence of OD correction with biological triplicates of sRef-bl and the medium. (PDF) [file pone.0224476.s006.pdf]

## Growth on fructose

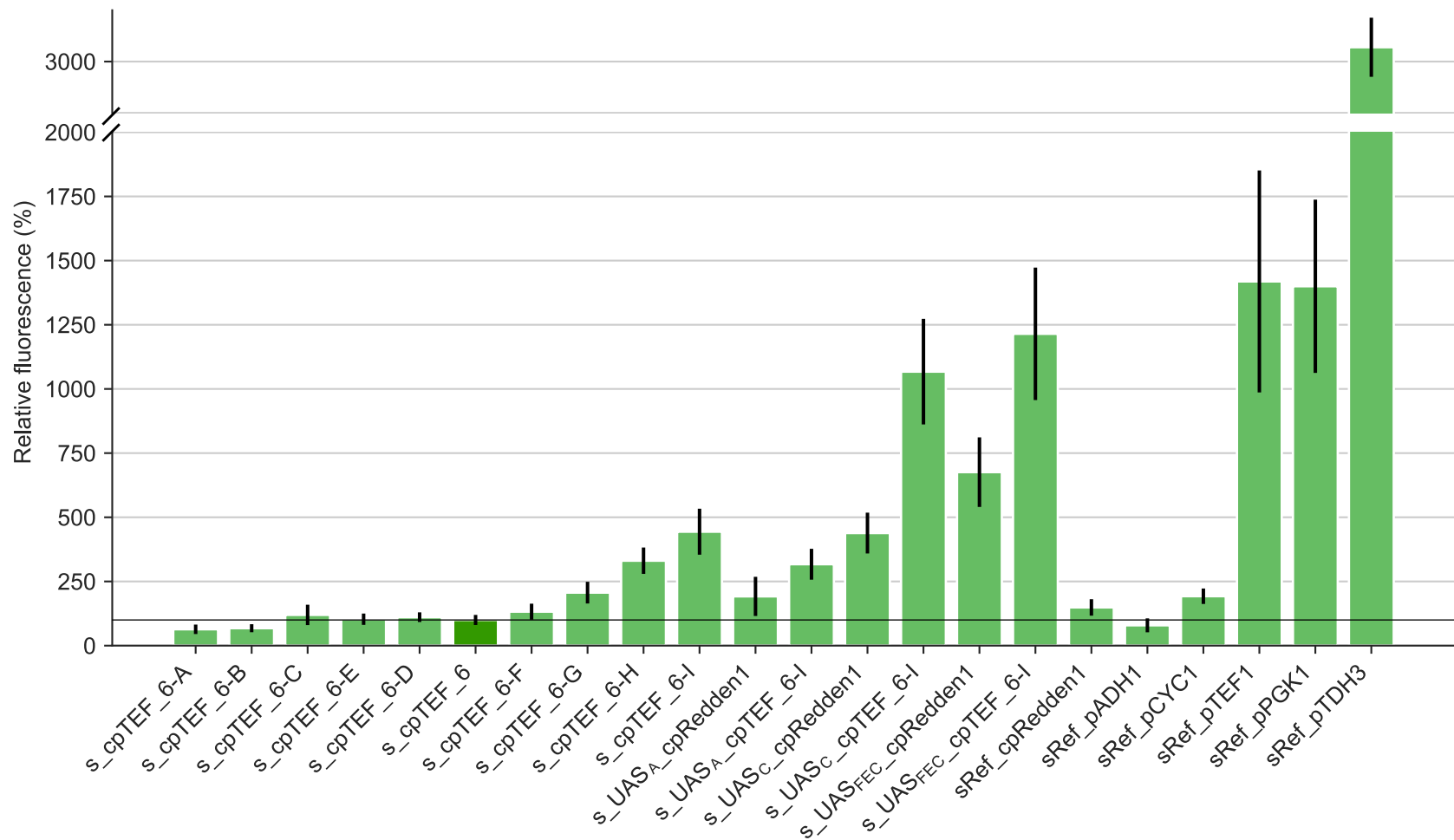

Supplement: S7 Fig — Protein expression levels were normalized against the native cpTEF_6 promoter (dark green, horizontal line). Error bars represent the standard error of the mean (n = 4, biological repeats). All strains are listed in Table A in S1 File. (PDF) [file pone.0224476.s007.pdf]

## Growth on pyruvate

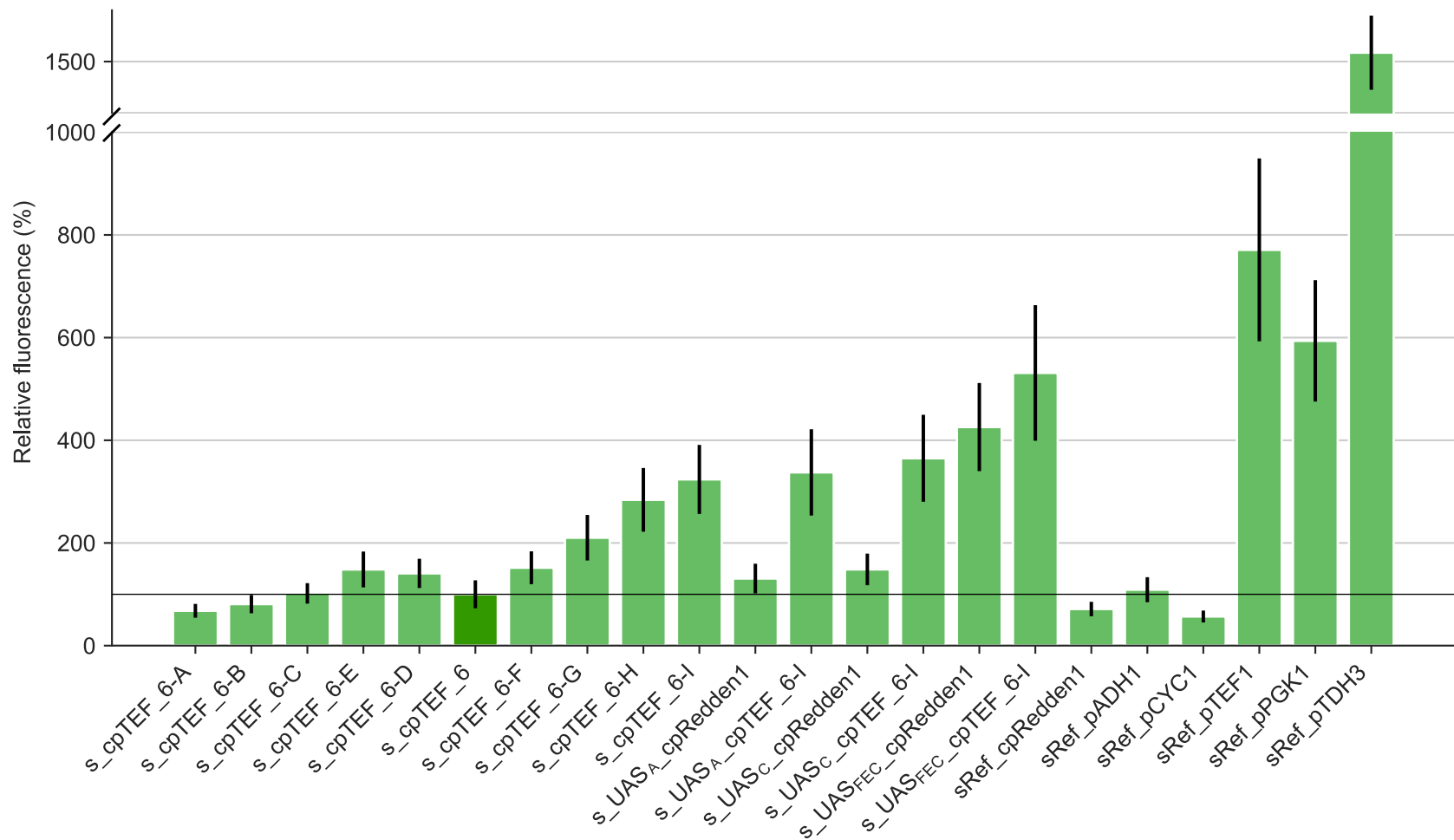

Supplement: S8 Fig — Protein expression levels were normalized against the native cpTEF_6 promoter (dark green, horizontal line). Error bars represent the standard error of the mean (n = 4, biological repeats). All strains are listed in Table A in S1 File. (PDF) [file pone.0224476.s008.pdf]

## Growth on glycerol

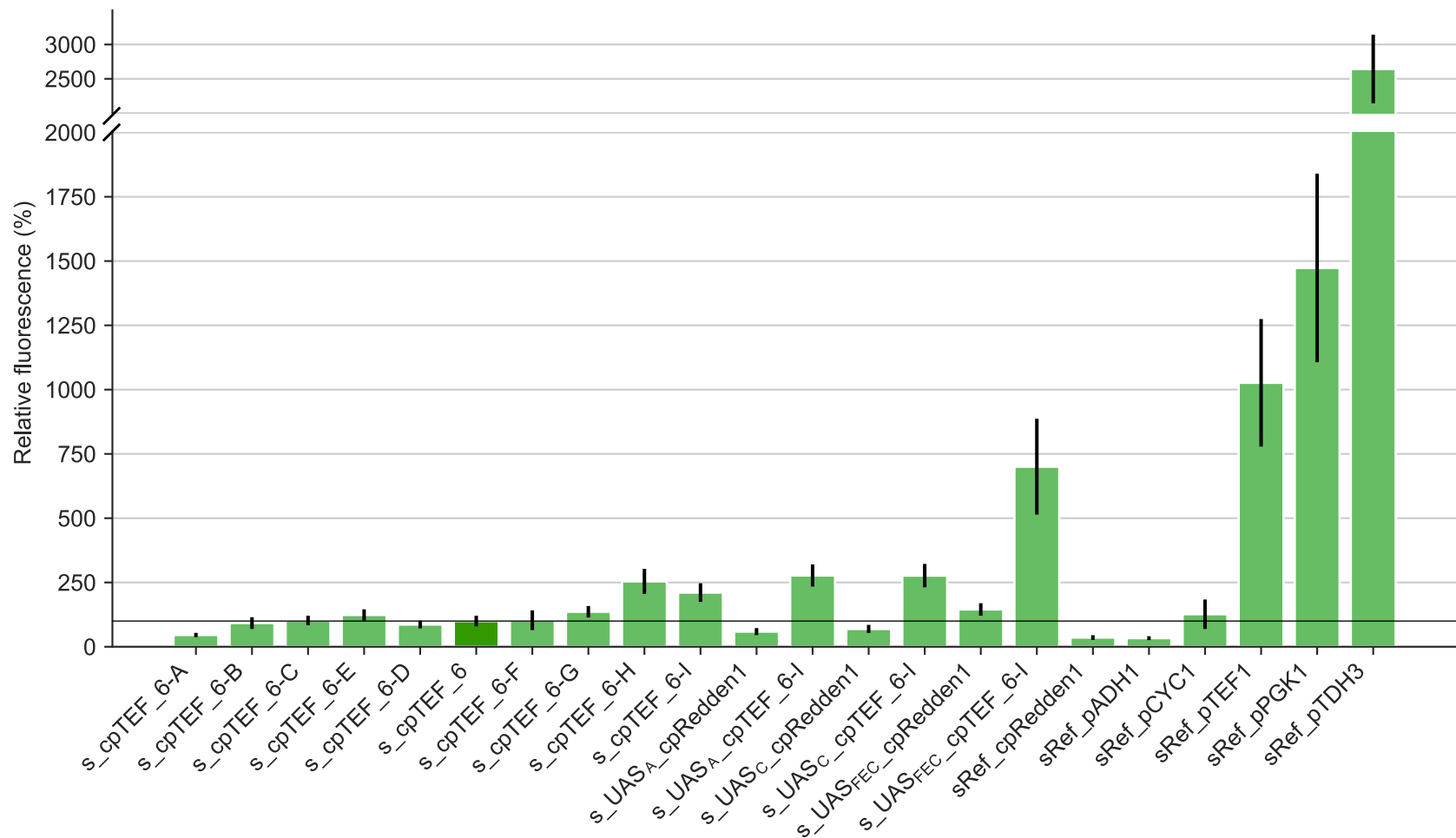

Supplement: S9 Fig — Protein expression levels were normalized against the native cpTEF_6 promoter (dark green, horizontal line). Error bars represent the standard error of the mean (n = 4, biological repeats). All strains are listed in Table A in S1 File. (PDF) [file pone.0224476.s009.pdf]
