# Supplementary material for: Modulating transcription through development of semi-synthetic yeast core promoters
Source: PLoS One. 2019 Nov 5;14(11):e0224476. doi: 10.1371/journal.pone.0224476 (PMC6830820; doi:10.1371/journal.pone.0224476)
Supplement: S1 File — Table A. Strains used in this study. Table B. Plasmids used in this study. Table C. Primers used in this study. Table D. Sequences of the core promoters. Table E. Synthetic upstream activating sequences. Table F. p-values obtained after one-way ANOVA to investigate the yECitrine variability of the native and synthetic promoters. (PDF) [file pone.0224476.s010.pdf]

Modulating transcription through development of semi-synthetic yeast core promoters

Supplemental tables

Thomas Decoene[1], Sofie L. De Maeseneire[2], Marjan De Mey[1*]

[1] Centre for Synthetic Biology (CSB), Ghent University, Coupure links 653, 9000 Ghent, Belgium

[2] Centre for Industrial Biotechnology and Biocatalysis (InBio.be), Ghent University, Coupure links 653, 9000 Ghent, Belgium

* Corresponding author:

E-mail: marjan.demey@ugent.be

Table A: Strains used in this study. All strains are obtained from strain SY992. In all plasmid and strain names, the *TEF1* promoter is shortly named as TEF and the core promoter as cpTEF. The genotype of the plasmids is listed in Table B in S1 File.

Strain	Genotype/Plasmid	Reference
SY992	*Matα, ura3Δ0, his3Δ1, leu2Δ0, trp1-63, ade2Δ0, lys2Δ0, ADE8*	Euroscarf (28)
sRef-pTEF1	pRef-pTEF1	This study
sRef-pADH1	pRef-pADH1	This study
sRef-pCYC1	pRef-pCYC1	This study
sRef-pPGK1	pRef-pPGK1	This study
sRef-pTDH3	pRef-pTDH3	This study
sRef-cpRedden1	pRef-cpRedden1	This study
sRef-bl	p2a_empty	This study
s_UAS-cpTEF_1	p_UAS-cpTEF_1 (pRef-pTEF1)	This study
s_UAS-cpTEF_2	p_UAS-cpTEF_2	This study
s_UAS-cpTEF_3	p_UAS-cpTEF_3	This study
s_UAS-cpTEF_4	p_UAS-cpTEF_4	This study
s_UAS-cpTEF_5	p_UAS-cpTEF_5	This study
s_UAS-cpTEF_6	p_UAS-cpTEF_6	This study
s_UAS-cpTEF_7	p_UAS-cpTEF_7	This study
s_UAS-cpTEF_8	p_UAS-cpTEF_8	This study
s_UAS-cpTEF_9	p_UAS-cpTEF_9	This study
s_cpTEF_1	p_cpTEF_1	This study
s_cpTEF_2	p_cpTEF_2	This study
s_cpTEF_3	p_cpTEF_3	This study
s_cpTEF_4	p_cpTEF_4	This study
s_cpTEF_5	p_cpTEF_5	This study
s_cpTEF_6	p_cpTEF_6	This study
s_cpTEF_7	p_cpTEF_7	This study
s_cpTEF_8	p_cpTEF_8	This study
s_cpTEF_9	p_cpTEF_9	This study
s_cpTEF_6-libA	p_cpTEF_6-libA	This study
s_cpTEF_6-libB	p_cpTEF_6-libB	This study
s_cpTEF_6-libC	p_cpTEF_6-libC	This study
s_cpTEF_6-libD	p_cpTEF_6-libD	This study
s_cpTEF_6-A	p_cpTEF_6-A	This study
s_cpTEF_6-B	p_cpTEF_6-B	This study
s_cpTEF_6-C	p_cpTEF_6-C	This study
s_cpTEF_6-D	p_cpTEF_6-D	This study
s_cpTEF_6-E	p_cpTEF_6-E	This study
s_cpTEF_6-F	p_cpTEF_6-F	This study
s_cpTEF_6-G	p_cpTEF_6-G	This study
s_cpTEF_6-H	p_cpTEF_6-H	This study
s_cpTEF_6-I	p_cpTEF_6-I	This study
s_UAS$_{A}$_cpTEF_6-I	p_UAS$_{A}$_cpTEF_6-I	This study
s_UAS$_{C}$_cpTEF_6-I	p_UAS$_{C}$_cpTEF_6-I	This study
s_UAS$_{FEC}$_cpTEF_6-I	p_UAS$_{FEC}$_cpTEF_6-I	This study

s_UAS$_{A}$_cpRedden1	p_UAS$_{A}$_cpRedden1	This study
s_UAS$_{C}$_cpRedden1	p_UAS$_{C}$_cpRedden1	This study
s_UAS$_{FEC}$_cpRedden1	p_UAS$_{FEC}$_cpRedden1	This study

Table B: Plasmids used in this study. All 5'UTRs are the native 5'UTR sequences of the preceding promoter. In all plasmid names, the *TEF1* promoter is shortly named as TEF and the core promoter as cpTEF.

Plasmid	Genotype/Description	Reference
pKT140	yECitrine-tADH1, *KAN*, AmpR, CEN/ARS	Euroscarf(29)
pRef-pTEF1	pTEF1-5'UTR-yECitrine-tADH1, *URA3*, AmpR, CEN/ARS	This study
pRef-pADH1	pADH1-5'UTR-yECitrine-tADH1, *URA3*, AmpR, CEN/ARS	This study
pRef-pCYC1	pCYC1-5'UTR-yECitrine-tADH1, *URA3*, AmpR, CEN/ARS	This study
pRef-pPGK1	pPGK1-5'UTR-yECitrine-tADH1, *URA3*, AmpR, CEN/ARS	This study
pRef-pTDH3	pTDH3-5'UTR-yECitrine-tADH1, *URA3*, AmpR, CEN/ARS	This study
pRef-cpRedden1	cpRedden1-5'UTR-yECitrine-tADH1, *URA3*, AmpR, CEN/ARS	This study
p2a_empty	*URA3*, CEN/ARS	This study
p_UAS-cpTEF_1	UAS-cpTEF_1-5'UTR-yECitrine-tADH1, *URA3*, AmpR, CEN/ARS	This study
p_UAS-cpTEF_2	UAS-cpTEF_2-5'UTR-yECitrine-tADH1, *URA3*, AmpR, CEN/ARS	This study
p_UAS-cpTEF_3	UAS-cpTEF_3-5'UTR-yECitrine-tADH1, *URA3*, AmpR, CEN/ARS	This study
p_UAS-cpTEF_4	UAS-cpTEF_4-5'UTR-yECitrine-tADH1, *URA3*, AmpR, CEN/ARS	This study
p_UAS-cpTEF_5	UAS-cpTEF_5-5'UTR-yECitrine-tADH1, *URA3*, AmpR, CEN/ARS	This study
p_UAS-cpTEF_6	UAS-cpTEF_6-5'UTR-yECitrine-tADH1, *URA3*, AmpR, CEN/ARS	This study
p_UAS-cpTEF_7	UAS-cpTEF_7-5'UTR-yECitrine-tADH1, *URA3*, AmpR, CEN/ARS	This study
p_UAS-cpTEF_8	UAS-cpTEF_8-5'UTR-yECitrine-tADH1, *URA3*, AmpR, CEN/ARS	This study
p_UAS-cpTEF_9	UAS-cpTEF_9-5'UTR-yECitrine-tADH1, *URA3*, AmpR, CEN/ARS	This study
p_cpTEF_1	cpTEF_1-5'UTR-yECitrine-tADH1, *URA3*, AmpR, CEN/ARS	This study
p_cpTEF_2	cpTEF_2-5'UTR-yECitrine-tADH1, *URA3*, AmpR, CEN/ARS	This study
p_cpTEF_3	cpTEF_3-5'UTR-yECitrine-tADH1, *URA3*, AmpR, CEN/ARS	This study
p_cpTEF_4	cpTEF_4-5'UTR-yECitrine-tADH1, *URA3*, AmpR, CEN/ARS	This study
p_cpTEF_5	cpTEF_5-5'UTR-yECitrine-tADH1, *URA3*, AmpR, CEN/ARS	This study
p_cpTEF_6	cpTEF_6-5'UTR-yECitrine-tADH1, *URA3*, AmpR, CEN/ARS	This study
p_cpTEF_7	cpTEF_7-5'UTR-yECitrine-tADH1, *URA3*, AmpR, CEN/ARS	This study
p_cpTEF_8	cpTEF_8-5'UTR-yECitrine-tADH1, *URA3*, AmpR, CEN/ARS	This study
p_cpTEF_9	cpTEF_9-5'UTR-yECitrine-tADH1, *URA3*, AmpR, CEN/ARS	This study
p_cpTEF_6-libA	cpTEF_6_libA-5'UTR-yECitrine-tADH1, *URA3*, AmpR, CEN/ARS	This study
p_cpTEF_6-libB	cpTEF_6_libB-5'UTR-yECitrine-tADH1, *URA3*, AmpR, CEN/ARS	This study
p_cpTEF_6-libC	cpTEF_6_libC-5'UTR-yECitrine-tADH1, *URA3*, AmpR, CEN/ARS	This study
p_cpTEF_6-libD	cpTEF_6_libD-5'UTR-yECitrine-tADH1, *URA3*, AmpR, CEN/ARS	This study
p_cpTEF_6-A	cpTEF_6-A-5'UTR-yECitrine-tADH1, *URA3*, AmpR, CEN/ARS	This study
p_cpTEF_6-B	cpTEF_6-B-5'UTR-yECitrine-tADH1, *URA3*, AmpR, CEN/ARS	This study
p_cpTEF_6-C	cpTEF_6-C-5'UTR-yECitrine-tADH1, *URA3*, AmpR, CEN/ARS	This study
p_cpTEF_6-D	cpTEF_6-D-5'UTR-yECitrine-tADH1, *URA3*, AmpR, CEN/ARS	This study
p_cpTEF_6-E	cpTEF_6-E-5'UTR-yECitrine-tADH1, *URA3*, AmpR, CEN/ARS	This study
p_cpTEF_6-F	cpTEF_6-F-5'UTR-yECitrine-tADH1, *URA3*, AmpR, CEN/ARS	This study
p_cpTEF_6-G	cpTEF_6-G-5'UTR-yECitrine-tADH1, *URA3*, AmpR, CEN/ARS	This study
p_cpTEF_6-H	cpTEF_6-H-5'UTR-yECitrine-tADH1, *URA3*, AmpR, CEN/ARS	This study
p_cpTEF_6-I	cpTEF_6-I-5'UTR-yECitrine-tADH1, *URA3*, AmpR, CEN/ARS	This study
p_UAS$_{A}$_cpTEF_6-I	UAS$_{A}$_cpTEF_6-I-5'UTR-yECitrine-tADH1, *URA3*, AmpR, CEN/ARS	This study
p_UAS$_{C}$_cpTEF_6-I	UAS$_{C}$_cpTEF_6-I-5'UTR-yECitrine-tADH1, *URA3*, AmpR, CEN/ARS	This study
p_UAS$_{FEC}$_cpTEF_6-I	UAS$_{FEC}$_cpTEF_6-I-5'UTR-yECitrine-tADH1, *URA3*, AmpR, CEN/ARS	This study
p_UAS$_{A}$_cpRedden1	UAS$_{A}$_cpRedden1-5'UTR-yECitrine-tADH1, *URA3*, AmpR, CEN/ARS	This study

p_UAS$_{C}$_cpRedden1	UAS$_{C}$_cpRedden1-5'UTR-yECitrine-tADH1, *URA3*, AmpR, CEN/ARS	This study
p_UAS$_{FEC}$_cpRedden1	UAS$_{FEC}$_cpRedden1-5'UTR-yECitrine-tADH1, *URA3*, AmpR, CEN/ARS	This study

Table C: Primers used in this study. In all plasmid names, the *TEF1* promoter is shortly named as TEF and the core promoter as cpTEF.

Primer N°	Sequence (5' – 3')	Description
1	ACTGTTGGGCGTGAGTGGAGGCGCCGGAAAAAAGCATCGAAAAA ATCTAGAAAAATGTCTAAAGGTGAAGAATTATTC	Construction primer for pRef-cpRedden1
2	TTTCCGGCGCCTCCACTCACGCCCAACAGTGCTCTTTTATAAGCT TGAATTCACTGGCCGTCGTTTTAC	Construction primer for pRef-cpRedden1
3	GAGACCGCCTCGTTTCTTTTTCTTCGTCGAAAAAGGCAATAAAAA TTTTTATCACGTTT	Construction primer for p_UAS-cpTEF_1
4	GAGACCGCCTCGTTTCTTTTTCTTCGTCGAAAAAGGCCTTTTTCT TGAAAATTTTTTTTTTTG	Construction primer for p_UAS-cpTEF_2
5	GAGACCGCCTCGTTTCTTTTTCTTCGTCGAAAAAGGCGATTTTTT TCTCTTTCGATGAC	Construction primer for p_UAS-cpTEF_3
6	GAGACCGCCTCGTTTCTTTTTCTTCGTCGAAAAAGGCGACCTCCC ATTGATATTTAAG	Construction primer for p_UAS-cpTEF_4
7	GAGACCGCCTCGTTTCTTTTTCTTCGTCGAAAAAGGCGTTAATAA ACGGTCTTCAATTTC	Construction primer for p_UAS-cpTEF_5
8	GAGACCGCCTCGTTTCTTTTTCTTCGTCGAAAAAGGCTCTCAAGT TTCAGTTTCATTTTTC	Construction primer for p_UAS-cpTEF_6
9	GAGACCGCCTCGTTTCTTTTTCTTCGTCGAAAAAGGCCTTGTTCT ATTACAACTTTTTTTAC	Construction primer for p_UAS-cpTEF_7
10	GAGACCGCCTCGTTTCTTTTTCTTCGTCGAAAAAGGCACTTCTTG CTCATTAGAAAGAAAG	Construction primer for p_UAS-cpTEF_8
11	GAGACCGCCTCGTTTCTTTTTCTTCGTCGAAAAAGGCAAGCATAG CAATCTAATCTAAG	Construction primer for p_UAS-cpTEF_9
12	CACGACGTTGTAAAACGACGGCCAGTGAATTCAATAAAAATTTT TATCACGTTTCTTTTTCTTG	Construction primer for p_cpTEF_1
13	CACGACGTTGTAAAACGACGGCCAGTGAATTCCTTTTTCTTGAAA ATTTTTTTTTTTG	Construction primer for p_cpTEF_2
14	CACGACGTTGTAAAACGACGGCCAGTGAATTCGATTTTTTTCTCT TTCGATGAC	Construction primer for p_cpTEF_3
15	CACGACGTTGTAAAACGACGGCCAGTGAATTCGACCTCCCATTGA TATTTAAG	Construction primer for p_cpTEF_4
16	CACGACGTTGTAAAACGACGGCCAGTGAATTCGTTAATAAACGG TCTTCAATTTC	Construction primer for p_cpTEF_5
17	CACGACGTTGTAAAACGACGGCCAGTGAATTCTCTCAAGTTTCAG TTTCATTTTTC	Construction primer for p_cpTEF_6
18	CACGACGTTGTAAAACGACGGCCAGTGAATTCCTTGTTCTATTAC AACTTTTTTTAC	Construction primer for p_cpTEF_7
19	CACGACGTTGTAAAACGACGGCCAGTGAATTCACTTCTTGCTCAT TAGAAAGAAAG	Construction primer for p_cpTEF_8
20	CACGACGTTGTAAAACGACGGCCAGTGAATTCAAGCATAGCAAT CTAATCTAAG	Construction primer for p_cpTEF_9
21	CGGCGTAATCATGGTCATAG	Forward primer to split p2a backbone
22	CGGATAACAATTTCACACAGG	Reverse primer to split p2a backbone
23	GCCTTTTTCGACGAAGAAAAAGAAACGAGGCGGTCTC	Reverse primer for construction p_cpTEF
24	GAATTCACTGGCCGTCGTTTTAC	Reverse primer for construction p_UAS-cpTEF
25	AGTAAAAAAAGTTGTAATAGAACAAGAAAAANNNNNNNNNNN NNNNNNNGAATTCACTGGCCGTCGTTTTACAACGTC	Construction primer for p_cpTEF_6-libA

26	CTTTCTAATGAGCAAGAAGTAAAAAAAGTTNNNNNNNNNNNNNNNNNNTGAAACTGAAACTTGAGAGAATTCAC	Construction primer for p_cpTEF_6-libB
27	GATTAGATTGCTATGCTTTCTTTCTAATGAGNNNNNNNNNNNNNNNNNNGTAATAGAACAAGAAAAATGAAAC	Construction primer for p_cpTEF_6-libC
28	CATTTTGTAATTAAAACTTAGATTAGATTGCTANNNNNNNNNNNNNNNNNNCAAGAAGTAAAAAAAGTTGTAATAG	Construction primer for p_cpTEF_6-libD
29	GCGGTGTTAATTAACTTGTAATATTCTAATCAAGCTAGATTTAAACTCGCGTGTTTTTTC	Construction primer for p_UAS$_{A}$_cpTEF_6-I
30	AATATTACAAGTTAATTAACACCGCCCCCGGCGCGCCGAATTCACTGGCCGTCGTTTTAC	Construction primer for p_UAS$_{A}$_cpTEF_6-I
31	ATGTGATTAATTAACTTGTAATATTCTAATCAAGCTAGATTTAAACTCGCGTGTTTTTTC	Construction primer for p_UAS$_{C}$_cpTEF_6-I
32	AATATTACAAGTTAATTAATCACATGCTAGGCGCGCCGAATTCACTGGCCGTCGTTTTAC	Construction primer for p_UAS$_{C}$_cpTEF_6-I
33	ATGTGATTAATTAACTTGTAATATTCTAATCAAGCTAGATTTAAACTCGCGTGTTTTTTC	Construction primer for p_UAS$_{FEC}$_cpTEF_6-I
34	AATATTACAAGTTAATTAATCACATGCTAAAATTTCAGTTTCAAGGAGGGGCGCGCCGAATTCACTGGCCGTCGTTTTAC	Construction primer for p_UAS$_{FEC}$_cpTEF_6-I
35	TGTAAAACGACGGCCAGTGAATTCGGCGCGCCGGGGGCGGTGTTAATTAACTTGTAATATTCTAATCAAGCTTATAAAAGAGCACTGTTG	Construction primer for p_UAS$_{A}$_cpRedden1
36	GAATTCACTGGCCGTCGTTTTAC	Construction primer for p_UAS$_{A}$_cpRedden1
37	TGTAAAACGACGGCCAGTGAATTCGGCGCGCCTAGCATGTGATTAATTAACTTGTAATATTCTAATCAAGCTTATAAAAGAGCACTGTTG	Construction primer for p_UAS$_{C}$_cpRedden1
38	GAATTCACTGGCCGTCGTTTTAC	Construction primer for p_UAS$_{C}$_cpRedden1
39	GGCGCGCCCCTCCTTGAAACTGAAATTTTAGCATGTGATTAATTAACTTGTAATATTCTAATCAAGCTTATAAAAGAGCACTGTTG	Construction primer for p_UAS$_{FEC}$_cpRedden1
40	CTAAAATTTCAGTTTCAAGGAGGGGCGCGCCGAATTCACTGGCCGTCGTTTTAC	Construction primer for p_UAS$_{FEC}$_cpRedden1

Table D: Sequences of the different core promoters obtained after truncation of the native 176 bp *TEF1* core promoter (cpTEF_1) and of the core promoter selection after randomization of the first 18 bp of cpTEF_6. The shortened sequence compared to the next cpTEF is indicated in bold. The modified core promoter part of cpTEF_6 after randomization and sequencing is indicated in bold and lowercase letters. TATA-like sequences of the strongest core promoters are underlined.

Core promoter	Sequence (5' – 3')
cpTEF_1 (176 bp)	**AATAAAAATTTTTATCACGTTT**CTTTTTCTTGAAAATTTTTTTTTTTGATTTTTTTTCTCTTTCGATGACCTCCCATTGATATTTAAGTTAATAAACGGTCTTCAATTTCTCAAGTTTCAGTTTCATTTTTCTTGTTCTATTACAACTTTTTTTACTTCTTGCTCATTAGAAAGAAA
cpTEF_2 (154 bp)	**CTTTTTCTTGAAAATTTTTTTTTTT**GATTTTTTTTCTCTTTCGATGACCTCCCATTGATATTTAAGTTAATAAACGGTCTTCAATTTCTCAAGTTTCAGTTTCATTTTTCTTGTTCTATTACAACTTTTTTTACTTCTTGCTCATTAGAAAGAAA
cpTEF_3 (129bp)	**GATTTTTTTTCTCTTTCGAT**GACCTCCCATTGATATTTAAGTTAATAAACGGTCTTCAATTTCTCAAGTTTCAGTTTCATTTTTCTTGTTCTATTACAACTTTTTTTACTTCTTGCTCATTAGAAAGAAA
cpTEF_4 (110 bp)	**GACCTCCCATTGATATTTAA**GTTAATAAACGGTCTTCAATTTCTCAAGTTTCAGTTTCATTTTTCTTGTTCTATTACAACTTTTTTTACTTCTTGCTCATTAGAAAGAAA
cpTEF_5 (90 bp)	**GTTAATAAACGGTCTTCAATTT**CTCAAGTTTCAGTTTCATTTTTCTTGTTCTATTACAACTTTTTTTACTTCTTGCTCATTAGAAAGAAA
cpTEF_6 (69 bp)	**TCTCAAGTTTCAGTTTCATTTTT**CTTGTTCTATTACAACTTTTTTTACTTCTTGCTCATTAGAAAGAAA
cpTEF_7 (46 bp)	**CTTGTTCTATTACAACTTTTTTT**ACTTCTTGCTCATTAGAAAGAAA
cpTEF_8 (23 bp)	**ACTTCTTGCTCATTAGAAAGA**AA
cpTEF_9 (2 bp)	AA
cpTEF_6-A	**tcggcacccagagacact**TTTTTCTTGTTCTATTACAACTTTTTTTACTTCTTGCTCATTAGAAAGAAA
cpTEF_6-B	**tccgccataaacgccgcc**TTTTTCTTGTTCTATTACAACTTTTTTTACTTCTTGCTCATTAGAAAGAAA
cpTEF_6-C	**cctcaacggaggcgtgtc**TTTTTCTTGTTCTATTACAACTTTTTTTACTTCTTGCTCATTAGAAAGAAA
cpTEF_6-D	**gaattaagagcacggccg**TTTTTCTTGTTCTATTACAACTTTTTTTACTTCTTGCTCATTAGAAAGAAA
cpTEF_6-E	**tatagtaatctaccttgc**TTTTTCTTGTTCTATTACAACTTTTTTTACTTCTTGCTCATTAGAAAGAAA
cpTEF_6-F	**tctcaagtttcagtttca**TTTTTCTTGTTCTATTACAACTTTTTTTACTTCTTGCTCATTAGAAAGAAA
cpTEF_6-G	**cactacatataatccgag**TTTTTCTTGTTCTATTACAACTTTTTTTACTTCTTGCTCATTAGAAAGAAA
cpTEF_6-H	**tacagccatatttaaatg**TTTTTCTTGTTCTATTACAACTTTTTTTACTTCTTGCTCATTAGAAAGAAA
cpTEF_6-I	**agatttaaactcgcgtgt**TTTTTCTTGTTCTATTACAACTTTTTTTACTTCTTGCTCATTAGAAAGAAA

Table E: Synthetic upstream activating sequences (UAS) together with the spacer sequence (26) which were used in front of cpTEF_6-I and cpRedden1. The UAS in indicated in bold, while the 30 bp spacer sequence is underlined.

UAS	Sequence (5' – 3')
UAS$_{A}$	**GGGGGCGGTG**TTAATTAACTTGTAATATTCTAATCAAGCT
UAS$_{C}$	**TAGCATGTGA**TTAATTAACTTGTAATATTCTAATCAAGCT
UAS$_{FEC}$	**CCTCCTTGAAACTGAAATTTTAGCATGTGA**TTAATTAACTTGTAATATTCTAATCAAGCT

Table F: The p-values obtained after one-way ANOVA to investigate the yECitrine variability of the native and synthetic promoters in the different growth experiments. In all cases, a significance level of 0.05 was applied.

Promoter	p-value	significant difference
pTEF1	0.106	No
pTDH3	3.16E-5	Yes
pPGK1	0.00915	Yes
pADH1	0.101	No
pCYC1	6.51E-5	Yes
cpRedden1	0.0393	Yes
cpTEF_6-I	0.979	No
